# Zero-Delay Multiple Descriptions of Stationary Scalar Gauss-Markov Sources

**DOI:** 10.3390/e21121185

**Published:** 2019-12-01

**Authors:** Andreas Jonas Fuglsig, Jan Østergaard

**Affiliations:** 1 Department of Electronic Systems, Aalborg University, 9000 Aalborg, Denmark; ajf@rtx.dk; 2 RTX A/S, 9400 Nørresundby, Denmark; 3 Section on Signal and Information Processing, Deparment of Electronic Systems, Aalborg University, 9000 Aalborg, Denmark

**Keywords:** zero delay, multiple descriptions, Gauss-Markov, source coding, rate distortion, feedback

## Abstract

In this paper, we introduce the zero-delay multiple-description problem, where an encoder constructs two descriptions and the decoders receive a subset of these descriptions. The encoder and decoders are causal and operate under the restriction of zero delay, which implies that at each time instance, the encoder must generate codewords that can be decoded by the decoders using only the current and past codewords. For the case of discrete-time stationary scalar Gauss—Markov sources and quadratic distortion constraints, we present information-theoretic lower bounds on the average sum-rate in terms of the directed and mutual information rate between the source and the decoder reproductions. Furthermore, we show that the optimum test channel is in this case Gaussian, and it can be realized by a feedback coding scheme that utilizes prediction and correlated Gaussian noises. Operational achievable results are considered in the high-rate scenario using a simple differential pulse code modulation scheme with staggered quantizers. Using this scheme, we achieve operational rates within 0.415 bits/sample/description of the theoretical lower bounds for varying description rates.

## 1. Introduction

Real-time communication is desirable in many modern applications, e.g., Internet of Things [1], audio transmission for hearing aids [2], stereo audio signals [3], on-line video conferencing [4], or systems involving feedback, such as networked control systems [5,6,7]. All these scenarios may operate under strict requirements on latency and reliability. Particularly, delays play a critical role in the performance or stability of these systems [8].

In near real-time communication over unreliable networks, and where retransmissions are either not possible or not permitted, e.g., due to strict latency constraints, it is generally necessary to use an excessive amount of bandwidth for the required channel code in order to guarantee reliable communications and ensure satisfactory performance. Several decades ago, it was suggested to replace the channel code by cleverly designed data packets, called multiple descriptions (MDs) [9]. Contrary to channel codes, MDs would allow for several reproduction qualities at the receivers and thereby admit a graceful degradation during partial network failures [9]. In MD coding, retransmissions are not necessary, which is similar to the case of forward error correction coding. Thus, with MDs, one avoids the possible long delay due to loss of packets or acknowledgement. Hence, some compression (reproduction quality) is sacrificed for an overall lower latency [9]. Interestingly, despite their potential advantages over channel codes for certain applications, MD codes are rarely used in practical communication systems with feedback. The reasons are that from a practical point of view, good MD codes are application-specific and hard to design, and from a theoretical point of view, zero-delay MD (ZDMD) coding and MD coding with feedback remain open and challenging topics.

### 1.1. Multiple Descriptions

MD coding can be described as a data compression methodology, where partial information about the data source is compressed into several data files (called descriptions or data packets) [10,11]. The descriptions can, for example, be individually transmitted over different channels in a network. The descriptions are usually constructed such that when any single description is decoded, it is possible to reconstruct an approximation of the original uncompressed source. Since this is only an approximation of the data source, there will inevitably be a reconstruction error, which yields a certain degree of distortion. The distinguishing aspect of MD coding over other coding methodologies is that if more than one description is retrieved, then a better approximation of the source is achieved than what is possible when only using a single description. As more descriptions are combined, the quality of the reproduced source increases. Similarly, this allows for a graceful degradation in the event of, e.g., packet dropouts on a packet-switched network such as the Internet.

**Figure 1:**
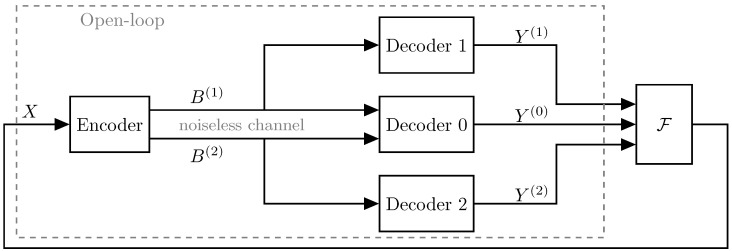
Multiple description (MD) source coding in a closed loop. If packet loss occurs on the noiseless channel, it will affect the source signal, *X*, differently depending on which descriptions are received. The standard open-loop MD coding is marked by the dashed line. In the open loop, the source is completely specified prior to the design of the coding scheme.

Figure 1 illustrates the two-description MD coding scenario in both a closed-loop and an open-loop system. In both cases, the encoder produces two descriptions which are transmitted across noiseless channels, i.e., no bit-errors are introduced in the descriptions between the encoder and decoders. Some work exists in the closed-loop scenario, but no complete solution has been determined. However, the noncausal open-loop problem has been more widely studied in the information-theory literature [9,10,11,12,13,14].

Since MD coding considers several data rates and distortions, MD rate-distortion theory is the determination of the fundamental limits on a rate-distortion region [9]. That is, determine the minimum individual rates required to achieve a given set of individual and joint distortion constraints. A noncausal achievable MD rate-distortion region is only completely known in very few cases [12]. El-Gamal and Cover [11] gave an achievable region for two descriptions and memoryless source. This region was then shown to be tight for white Gaussian sources with mean-squared error (MSE) distortion constraints by Ozarow [10]. In the high resolution limit, i.e., high rates, the authors of [13] characterized the achievable region for stationary (time-correlated) Gaussian sources with MSE distortion constraints. This was then extended in [14] to the general resolution case for stationary Gaussian sources. Recently, the authors of [12] showed in the symmetric case, i.e., equal rates and distortions for each individual description, that the MD region for a colored Gaussian source subject to MSE distortion constraints can be achieved by predictive coding using filtering. However, similar to single-description source coding [8], the MD source coders whose performance is close to the fundamental rate-distortion bounds impose long delays on the end-to-end processing of information, i.e., the total delay only due to source coding [15].

### 1.2. Zero Delay

Clearly, in near real-time communication, the source encoder and decoder must have zero delay. The term zero-delay (ZD) source coding is often used when both instantaneous encoding and decoding are required [16]. That is, when the reconstruction of each input sample must take place at the same time-instant, the corresponding input sample has been encoded [17]. For near instantaneous coding, the source coders must be causal [18]. However, causality comes with a price. The results of [17] showed that causal coders increase the bit-rate due to the space-filling loss of “memoryless” quantizers, and the reduced de-noising capabilities of causal filters. Additionally, imposing ZD increases the bit-rate due to memoryless entropy coding [17].

In the single-description case, ZD rate-distortion theory has been increasingly more popular in recent decades, due to its significance in real-time communication systems and especially feedback systems. Some indicative results on ZD source coding for networked control systems and systems with and without feedback may be found in [5,6,7,8,17,19,20,21]. The results of [5] establish a novel information-theoretic lower bound on the average data-rate for a source coding scheme within a feedback loop by the directed information rate across the channel. For open-loop vector Gauss-Markov sources, i.e., when the source is not inside a feedback loop, the optimal operational performance of a ZD source code subject to an MSE distortion constraint has been shown to be lower bounded by a minimization of the directed information [22] from the source to the reproductions subject to the same distortion constraint [5,6,7,17,19]. For Gaussian sources, the directed information is further minimized by Gaussian reproductions [8,20]. Very recently, Stavrou et al. [8], extending upon the works of [6,7,17,19], showed that the optimal test channel that achieves this lower bound is realizable using a feedback realization scheme. Furthermore, Ref [8] extended this to a predictive coding scheme providing an achievable upper bound on the operational performance subject to an MSE distortion constraint.

### 1.3. Zero-Delay Multiple Descriptions

Recently, the authors of [15] proposed an analog ZDMD joint source-channel coding scheme, such that the analog source output is mapped directly into analog channel inputs, thus not suffering from the delays encountered in digital source coding. However, for analog joint source-channel coding to be effective, the source and channel must be matched, which rarely occurs in practice [23]. Furthermore, most modern communication systems rely on digital source coding. Thus, analog joint source-channel coding is only applicable in a very limited amount of settings. Digital low-delay MD coding for practical audio transmission has been explored in, e.g., [2,4,24], as well as for low-delay video coding in [25]. Some initial work regarding MDs in networked control systems may be found in [26]. However, none of these consider the theoretical limitations of ZDMD coding in a rate-distortion sense.

In this paper, we propose a combination of ZD and MD rate-distortion theory such that the MD encoder and decoders are required to be causal and of zero delay. For the case of discrete-time stationary scalar Gauss-Markov sources and quadratic distortion constraints, we present information-theoretic lower bounds on the average sum-rate in terms of the directed and mutual information rate between the source and the decoder reproductions. We provide proof of achievability via a new Gaussian MD test channel and show that this test channel can be realized by a feedback coding scheme that utilizes prediction and correlated Gaussian noises. We finally show that a simple scheme using differential pulse code modulation with staggered quantizers can get close to the optimal performance. Specifically, our simulation study reveals that for a wide range of description rates, the achievable operational rates are within 0.415 bits/sample/description of the theoretical lower bounds. Further simulations and more details regarding the combination of ZD and MD coding are provided in the report [27].

The rest of the paper is organized as follows. In Section 2, we characterize the ZDMD source coding problem with feedback for stationary scalar Gauss-Markov sources subject to asymptotic MSE distortion constraints. Particularly, we consider the symmetric case in terms of the symmetric ZDMD rate-distortion function (RDF). In Section 3, we introduce a novel information-theoretic lower bound on the average data sum-rate of a ZDMD source code. For scalar stationary Gaussian sources, we show this lower bound is minimized by jointly Gaussian MDs, given that certain technical assumptions are met. This provides an information-theoretic lower bound to the symmetric ZDMD RDF. In Section 4, we determine an MD feedback realization scheme for the optimum Gaussian test-channel distribution. Utilizing this, we present a characterization of the Gaussian achievable lower bound as a solution to an optimization problem. In Section 5, we evaluate the performance of an operational staggered predictive quantization scheme compared to the achievable ZDMD region. We then discuss and conclude on our results. Particularly, we highlight some important difficulties with the extension to the Gaussian vector case.

## 2. Problem Definition

**Figure 2:**
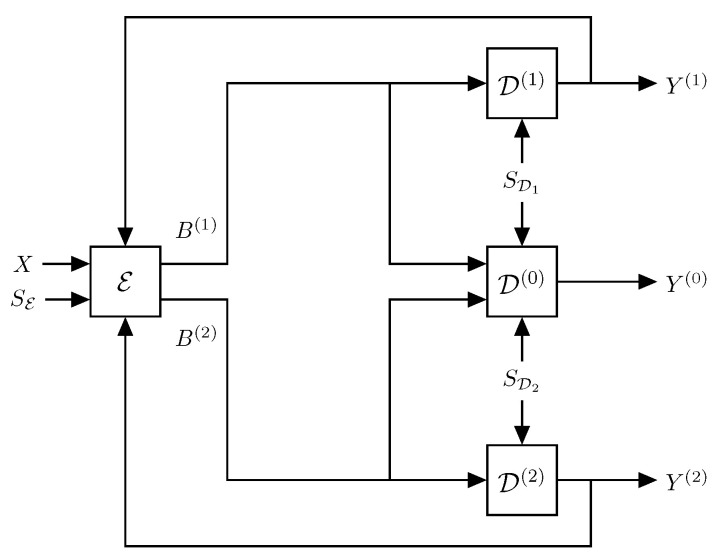
A general MD source-coding scenario with feedback.

In this paper, we consider the ZDMD source coding problem with feedback illustrated in Figure 2. The feedback channels are assumed to be noiseless digital channels and have a one-sample delay to ensure the operational feasibility of the system, i.e., at any time, the current encoder outputs only depend on previous decoder outputs.

Here, the stationary scalar Gauss-Markov source process is determined by the following discrete-time linear time-invariant model:

(1) $$X_{k+1}=aX_{k}+W_{k},\;k\in N,$$

where |a|<1 is the deterministic correlation coefficient, $X_{1}\in R∼N\left(0,\sigma_{X_{1}}^{2}\right)$ is the initial state, $\sigma_{X_{1}}^{2}=\frac{\sigma_{W}^{2}}{1-a^{2}}$, and $W_{k}\in R∼N\left(0,\sigma_{W}^{2}\right)$ is an independent and identically distributed (IID) Gaussian process independent of $\left\{X_{k}:k\in N\right\}$. For each time step k∈N, the ZDMD encoder, E, observes a new source sample $X_{k}$ while assuming it has already observed the past sequence $X^{k-1}$. The encoder then produces two binary descriptions $B_{k}^{\left(1\right)},B_{k}^{\left(2\right)}$ with lengths $l_{k}^{\left(1\right)},\;l_{k}^{\left(2\right)}$ (in bits) from two predefined sets of codewords $B_{k}^{\left(1\right)},B_{k}^{\left(2\right)}$, of at most a countable number of codewords, i.e., the codewords are discrete random variables. The codewords are transmitted across two instantaneous noiseless digital channels to the three reconstruction decoders, $D^{\left(0\right)},D^{\left(1\right)}$, and $D^{\left(2\right)}$. The decoders then immediately decode the binary codewords. Upon receiving $B^{(i),k}$, the *i*th side decoder, $D^{\left(i\right)}$, i=1,2, produces an estimate $Y_{k}^{\left(i\right)}$ of the source sample $X_{k}$, under the assumption that $Y^{(i),k-1}$ is already produced. Similarly, the central decoder, $D^{\left(0\right)}$, upon receiving $B^{(1),k},B^{(2),k}$, produces an estimate $Y_{k}^{\left(0\right)}$ of $X_{k}$ under the assumption $Y^{(0),k}$ is already produced. Finally, before generating the current binary codewords, the encoder receives the two reproductions from the previous time step $Y_{k-1}^{\left(1\right)},Y_{k-1}^{\left(2\right)}$ while assuming it has already received the past, $Y^{(1),k-2},\;Y^{(2),k-2}$.

We assume the encoder and all decoders process information without delay. That is, each sample is processed immediately and without any delays for each time step k∈N.

In the system, $S_{E,k}$ is the side information that becomes available at time-instance *k* at the encoder, and similarly, $S_{D_{i},k}$ is the new side information at reproduction decoder *i*. We emphasize, this is not side information in the usual information-theoretic sense of multiterminal source coding or Wyner–Ziv source coding, where the side information is unknown, jointly distributed with the source, and only available at the decoder, e.g., some type of channel-state information [28,29]. In this paper, our encoders and decoders are deterministic. However, to allow for probabilistic encoders and decoders, we let the deterministic encoders and decoders depend upon a stochastic signal, which we refer to as the side information. To make the analysis tractable, we require this side information to be independent of the source. The side information could, for example, represent dither signals in the quantizers, which is a common approach in the source coding literature [30]. We shortly disucuss the possibility of removing this independence assumption in Section 6.

We do not need feedback from the central decoder, since all information regarding $Y^{(0),k-1}$ is already contained in $\left(Y^{(1),k-1},Y^{(2),k-1}\right)$. That is, given the side information, the side decoder reproductions are sufficient statistics for the central reproduction, and the following Markov chain holds,

(2) $$X^{k}{|}_{\phi }-\left(Y^{(1),k},Y^{(2),k}\right){|}_{\phi }-Y^{(0),k}{|}_{\phi },$$

where $\phi =\left(S_{D_{1}}^{k},S_{D_{2}}^{k}\right)$. We note, this Markov chain also requires the decoders are invertible as defined in Definition 5 on page 10. Requiring invertible decoders is optimal in causal source coding [5].

**Figure 3:**
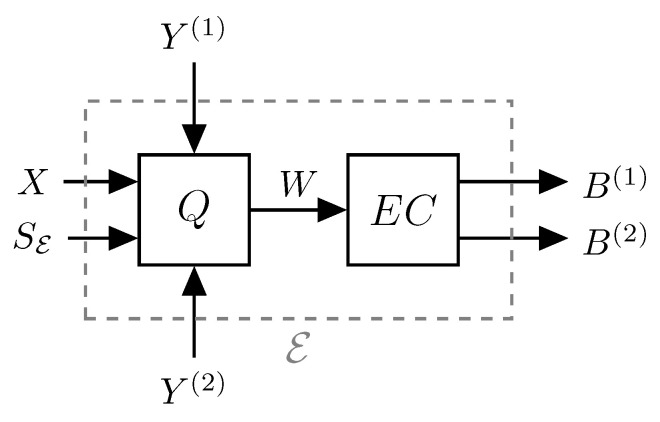
Conceptual model of splitting the zero-delay MD (ZDMD) encoder, E, into a lossy quantizer, *Q*, and a lossless entropy coder, EC. *W* is a *p*-dimensional signal, where *p* is appropriately chosen according to the employed quantization procedure.

***Zero-delay multiple-description source coding with side information:*** We specify in detail the operations of the different blocks in Figure 2. First, at each time step, *k*, all source samples up to time *k*, $X^{k}$, and all previous reproductions, $Y^{(i),k-1},\;i=1,2$, are available to the encoder, E. The encoder then performs lossy source coding and lossless entropy coding to produce two dependent codewords. That is, the encoder block can be conceptualized as being split into a quantization step and an entropy coding step as illustrated in Figure 3. This is a very simplified model, and each of the quantization and entropy coding steps may be further decomposed as necessary to generate the appropriate dependent messages. However, this is a nontrivial task, and therefore, for a more tractable analysis and ease of reading, we do not further consider this two-step procedure in the theoretical derivations.

The zero-delay encoder is specified by the sequence of functions $\left\{E_{k}:k\in N\right\}$, where:

(3) $$E_{k}:X^{k}\times Y^{(1),k-1}\times Y^{(2),k-1}\times S_{E}^{k}\to B_{k}^{\left(1\right)}\times B_{k}^{\left(2\right)},$$

and at each time step k∈N, the encoder outputs the messages:

(4) $$\begin{array}{cc} \left(B_{k}^{\left(1\right)},\;B_{k}^{\left(2\right)}\right) & =E_{k}\left(X^{k},Y^{(1),k-1},Y^{(2),k-1},S_{E}^{k}\right),\;k\in N, \end{array}$$

with length $l_{k}^{\left(i\right)}\;i=1,2$ (in bits), where for the initial encoding, there are no past reproductions available at the encoder, hence $\left(B_{1}^{\left(1\right)},B_{1}^{\left(2\right)}\right)=E_{1}\left(X_{1},S_{E,1}\right)$.

The zero-delay decoders are specified by the three sequences of functions $\left\{D_{k}^{\left(0\right)},D_{k}^{\left(1\right)},D_{k}^{\left(2\right)}:k\in N\right\}$, where:

(5) $$\begin{array}{cc} D_{k}^{\left(i\right)} & :B^{(i),k}\times S_{D_{i}}^{k}\to Y_{k}^{\left(i\right)},\;i=1,2, \end{array}$$

(6) $$\begin{array}{cc} D_{k}^{\left(0\right)} & :B^{(1),k}\times B^{(2),k}\times S_{D_{1}}^{k}\times S_{D_{2}}^{k}\to Y_{k}^{\left(0\right)}. \end{array}$$

At each time step, k∈N the decoders generate the outputs:

(7) $$\begin{array}{cc} Y_{k}^{\left(i\right)} & =D_{k}^{\left(i\right)}\left(B^{(i),k},S_{D_{i}}^{k}\right),\;i=1,2, \end{array}$$

(8) $$\begin{array}{cc} Y_{k}^{\left(0\right)} & =D_{k}^{\left(0\right)}\left(B^{(1),k},B^{(2),k},S_{D_{1}}^{k},S_{D_{2}}^{k}\right), \end{array}$$

assuming $Y^{(i),k-1},\;i=0,1,2$ have already been generated, with:

(9) $$\begin{array}{cc} Y_{1}^{\left(i\right)} & =D_{1}^{\left(i\right)}\left(B_{1}^{\left(i\right)},S_{D_{i},1}\right),\;i=1,2, \end{array}$$

(10) $$\begin{array}{cc} Y_{1}^{\left(0\right)} & =D_{1}^{\left(0\right)}\left(B_{1}^{\left(1\right)},B_{1}^{\left(2\right)},S_{D_{1},1},S_{D_{2},1}\right). \end{array}$$

The ZDMD source code produces two descriptions of the source; hence, we may associate the ZDMD code with a rate pair.

**Definition** **1** (Rate pair of ZDMD code)**.** *For each time step, k, let* $l_{k}^{\left(i\right)}$ *be the length in bits of the ith encoder output in a ZDMD source code as described above. Then, the average expected data-rate pair,* $\left(R_{1},\;R_{2}\right)$*, measured in bits per source sample, are the rates:*

(11) $$\begin{array}{c} R_{i}=\lim_{n\to \infty }\frac{1}{n}\sum_{k=1}^{n}E\left[l_{k}^{\left(i\right)}\right],\;i=1,2. \end{array}$$

***Asymptotic MSE distortion constraints:*** A rate pair $\left(R_{1},R_{2}\right)$ is said to be achievable with respect to the MSE distortion constraints $D_{i}>0,\;i=0,1,2,$ if there exists a rate-$\left(R_{1},R_{2}\right)$ ZDMD source code as described above, such that:

(12) $$\lim_{n\to \infty }\frac{1}{n}\sum_{k=1}^{n}E\left[{\left(X_{k}-Y_{k}^{\left(i\right)}\right)}^{2}\right]\leq D_{i},\;i=0,1,2,$$

is satisfied.

Similarly to standard MD theory [31], the main concern of ZDMD coding is to determine the ZDMD rate-region, constituting the set of all achievable rate pairs for given distortion constraints.

**Definition** **2** (ZDMD rate-region)**.** *For the stationary source process* $\left\{X_{k}\right\},\;X_{k}\in X$*, the ZDMD rate-region* $R_{X}^{ZD}\left(R_{1},R_{2},D_{0},D_{1},D_{2}\right)$ *is the convex closure of all achievable ZDMD rate pairs* $\left(R_{1},R_{2}\right)$ *with respect to the MSE distortion constraints* $\left(D_{0},D_{1},D_{2}\right)$*.*

The ZDMD rate-region can be fully characterized by determining the bound between the sets of achievable and non-achievable rates, i.e., by determining the fundamental smallest achievable rates for given distortion constraints. Particularly, we consider so-called nondegenerate distortion constraints [32], that is, triplets $\left(D_{0},D_{1},D_{2}\right)$ that satistify:

(13) $$\begin{array}{c} D_{1}+D_{2}-\sigma_{X}^{2}\leq D_{0}\leq {\left(\frac{1}{D_{1}}+\frac{1}{D_{2}}-\frac{1}{\sigma_{X}^{2}}\right)}^{-1}, \end{array}$$

where $\sigma_{X}^{2}$ is the stationary variance of the source.

The previous design requirements are summarized in the ZDMD coding problem with feedback.

**Problem** **1** (ZDMD coding problem with feedback)**.** *For a discrete-time stationary scalar source process* $\left\{X_{k}\right\}$*, with nondegenerate MSE distortion constraints,* $D_{0},D_{1},D_{2}>0$*. Determine the minimum operational rates* $R_{1},R_{2}$ *of the ZDMD coding scheme with side information from Equations* (3)–(8)*, such that the asymptotic average expected distortions satisfy:*

(14) $$\begin{array}{cc} \lim_{n\to \infty }\frac{1}{n}\sum_{k=1}^{n}E\left[{\left(X_{k}-Y_{k}^{\left(i\right)}\right)}^{2}\right] & \leq D_{i},\;i=0,1,2. \end{array}$$

*where the minimum is over all possible ZDMD encoder and decoder sequences* ${\left\{E_{k}\right\}}_{k\in N},\;{\left\{D_{k}^{\left(i\right)}\right\}}_{k\in N},\;i=0,1,2$ *that satisfy Equations* (3)–(8)*.*

In this paper, we mainly consider the symmetric case of $R_{1}=R_{2}=R$ and $D_{1}=D_{2}=D_{S}$. Here, the ZDMD region may be completely specified by an MD equivalent of the standard RDF [12].

**Definition** **3** (Symmetric ZDMD RDF)**.** *The symmetric ZDMD RDF for a source,* {X}*, with MSE distortion constraints,* $D_{0},\;D_{S}>0$*, is:*

(15) $$\begin{array}{ccc} R_{ZD}^{op}\left(D_{0},D_{S}\right)≜ & \inf & R\\ \; & s.t. & \left(R,R\right)\in R_{X}^{ZD}\left(R,R,D_{0},D_{S},D_{S}\right). \end{array}$$

*That is the minimum rate R per description, which is achievable with respect to the distortion pair* $\left(D_{0},D_{S}\right)$ *.*

The operational symmetric ZDMD RDF can be expressed in terms of the sum-rate, $R_{1}+R_{2}$.

**Problem** **2** (Operational symmetric scalar Gaussian ZDMD RDF)**.** *For a stationary scalar Gauss-Markov source process* (1)*, with nondegenerate MSE distortion constraints,* $D_{0},\;D_{S}>0$*, determine the operational symmetric ZDMD RDF, i.e., solve the optimization problem:*

(16) $$\begin{array}{ccc} R_{ZD}^{op}\left(D_{0},D_{S}\right) & =\inf\; & \lim_{n\to \infty }\frac{1}{2n}\left(E\left[L_{n}^{\left(1\right)}\right]+E\left[L_{n}^{\left(2\right)}\right]\right)\\ & s.t. & \lim_{n\to \infty }\frac{1}{n}\sum_{k=1}^{n}E\left[{\left(X_{k}-Y_{k}^{\left(0\right)}\right)}^{2}\right]\leq D_{0}\\ & & \lim_{n\to \infty }\frac{1}{n}\sum_{k=1}^{n}E\left[{\left(X_{k}-Y_{k}^{\left(i\right)}\right)}^{2}\right]\leq D_{S},\;i=1,2, \end{array}$$

*where* $L_{n}^{\left(i\right)}≜\sum_{k=1}^{n}l_{k}^{\left(i\right)},\;i=1,2,$ *and the infimum is over all possible ZDMD encoder- and -decoder sequences* ${\left\{E_{n}\right\}}_{n\in N},\;{\left\{D_{n}^{\left(0\right)}\right\}}_{n\in N},\;{\left\{D_{n}^{\left(1\right)}\right\}}_{n\in N},\;{\left\{D_{n}^{\left(2\right)}\right\}}_{n\in N}$*, i.e., that satisfy Equations* (3)–(8)*.*

Unfortunately, the solutions to Problems 1 and 2 are very hard to find, since they are determined by a minimization over all possible operational ZDMD codes. Similar to single description ZD rate-distortion theory [17], where the classical RDF is a lower bound on the zero-delay RDF, the noncausal arbitrary delay MD region [10,14] is an outer bound on the ZDMD region. However, this is a conservative bound due to the space-filling losses, memoryless entropy coding, and causal filters suffered by the ZD coders. Therefore, we introduce a novel information-theoretic lower bound on the operational ZD coding rates. As in classical MD rate-distortion theory, this bound is given in terms of lower bounds on the marginal rates, $R_{1},\;R_{2}$, and the sum-rate, $R_{1}+R_{2}$ cf. [10,11].

## 3. Lower Bound on Average Data-Rate

In this section, we determine a novel information-theoretic lower bound on the sum-rate of ZDMD source coding with feedback. Using this lower bound, we present an information-theoretic counterpart of the operational symmetric Gaussian ZDMD RDF. Finally, we provide a lower bound to Problem 2 by showing, for stationary scalar Gaussian sources, that Gaussian reproductions minimize the information-theoretic lower bound, given some technical assumptions are met. Although our main concern is the symmetric case, some of our main results are provided in the general nonsymmetric case.

We study a lower bound on the sum-rate of the ZDMD coding problem with feedback, which only depends on the joint statistics of the source encoder input, *X*, and the decoder outputs, $Y^{\left(i\right)}\;i=0,1,2$. To this end, we present in more detail the test-channel distribution associated with this minimization.

### 3.1. Distributions

We consider a source that generates a stationary sequence $X_{k}=x_{k}\in X_{k},\;k\in N^{n}$. The objective is to reproduce or reconstruct the source by $Y_{k}^{\left(i\right)}=y_{k}^{\left(i\right)}\in Y_{k}^{\left(i\right)},\;k\in N^{n},\;i=0,1,2$, subject to MSE fidelity criteria $d_{1,n}^{\left(i\right)}\left(x^{n},y^{(i),n}\right)≜\frac{1}{n}\sum_{k=1}^{n}{\left(x_{k}-y_{k}^{\left(i\right)}\right)}^{2},\;i=0,1,2$.

*Source*. We consider open-loop source coding; hence, we assume the source distribution satisfies the following conditional independence:

(17) $$P\left(x_{k}|x^{k-1},y^{(0),k-1},y^{(1),k-1},y^{(2),k-1}\right)≜P\left(x_{k}|x^{k-1}\right),\;k\in N^{n}.$$

This implies that the source, *X*, is unaffected by the feedback from the reproductions, $Y^{\left(i\right)}$. Hence, the next source symbol, given the previous symbols, is not further related to the previous reproductions [22].

We assume the distribution at k=1 is $P(x_{1})$. Furthermore, by Bayes’ rule [8]:

(18) $$P\left(x^{n}\right)≜\prod_{k=1}^{n}P\left(x_{k}|x^{k-1}\right).$$

For the Gauss-Markov source process (1), this implies $\left\{W_{k}\right\}$ is independent of the past reproductions $Y^{(i),k-1},\;i=0,1,2$ [8].

*Reproductions*. Since the source is unaffected by the feedback from the reproductions, the MD encoder–decoder pairs from E to $D_{i},\;i=0,1,2$, in Figure 2, are causal if, and only if, the following Markov chain holds [17]:

(19) $$X_{k+1}^{n}-X^{k}-\left(Y^{(0),k},Y^{(1),k},Y^{(2),k}\right),\;\forall k\in \left\{1,\ldots ,n-1\right\}.$$

Hence, we assume the reproductions are randomly generated according to the collection of conditional distributions:

(20) $$P\left(y_{k}^{\left(0\right)},y_{k}^{\left(1\right)},y_{k}^{\left(2\right)}|y^{(0),k-1},y^{(1),k-1},y^{(2),k-1},x^{k}\right),\;k\in N^{n}.$$

For the first time step, k=1, we assume:

(21) $$P\left(y_{1}^{\left(0\right)},y_{1}^{\left(1\right)},y_{1}^{\left(2\right)}|y^{(0),0},y^{(1),0},y^{(2),0},x^{1}\right)=P\left(y_{1}^{\left(0\right)},y_{1}^{\left(1\right)},y_{1}^{\left(2\right)}|x_{1}\right).$$

### 3.2. Bounds

We define the directed information rate across a system with random input and random output processes.

**Definition** **4** (Directed information rate ([5] Def. 4.3))**.** *The directed information rate across a system with random input, X, and random output, Y, is defined as:*

(22) $$\begin{array}{cc} \bar{I}\left(X\to Y\right) & ≜\lim_{n\to \infty }\frac{1}{n}I\left(X^{n}\to Y^{n}\right) \end{array}$$

*where* $I\left(X^{n}\to Y^{n}\right)$ *is the directed information between the two sequences* $X^{n}$ *and* $Y^{n}$*, defined as:*

(23) $$\begin{array}{c} I\left(X^{n}\to Y^{n}\right)≜\frac{1}{n}\sum_{k=1}^{n}I\left(X^{k};Y_{k}|Y^{k-1}\right). \end{array}$$

In order to establish an outer bound on the ZDMD rate-region, we need a lower bound on the marginal rates and the sum-rate. By the results of [5,8], it can be shown that the marginal operational rates, $R_{1},R_{2}$ are lower bounded by:

(24) $$\begin{array}{cc} R_{i} & \geq \bar{I}\left(X\to Y^{\left(i\right)}\right), \end{array}$$

(25) $$\begin{array}{cc} \; & =\lim_{n\to \infty }\frac{1}{n}I\left(X^{n}\to Y^{(i),n}\right), \end{array}$$

(26) $$\begin{array}{cc} \; & =\lim_{n\to \infty }\frac{1}{n}\sum_{k=1}^{n}I\left(X^{k};Y_{k}^{\left(i\right)}|Y^{(i),k-1}\right),\;i=1,2, \end{array}$$

that is, by the directed information rate from the source to the side description. Thus, in order to determine a bound on the ZDMD rate-region, it remains to determine an information-theoretic lower bound on the sum-rate. Our derivation of the lower bound on the sum-rate requires the following assumption.

**Assumption** **1.** *The systems*$E,D^{\left(i\right)}$*i = 0,1,2, are causal, described by Equations* (3)–(8)*, and* $\left(\left\{S_{D_{1}}\right\},\left\{S_{D_{2}}\right\}\right)⫫\left\{X_{k}\right\}$*, i.e., the side information is independent of the source sequence,* $\left\{X_{k}\right\}$*.*

We consider this assumption to be reasonable in a ZD scenario, i.e., the deterministic encoders and decoders must be causal and use only past and present symbols, and side information that is not associated with the source signal [5]. Similar to [5], the channel is the only link between encoder and decoder. However, we further assume the channel to have perfect feedback.

Additionally, we require the decoders to be invertible given the side information.

**Definition** **5**  (Invertible decoder ([5] Def. 4.2))**.** *The decoders,* $D^{\left(i\right)},\;i=0,1,2$*, defined in Equations* (7) *and* (8) *are said to be invertible if, and only if,* ∀k∈N*, there exists deterministic mappings* $G_{k}^{\left(i\right)},\;i=0,1,2$*, such that:*

(27) $$\begin{array}{cc} B^{(1),k} & =G_{k}^{\left(1\right)}\left(Y_{k}^{\left(1\right)},S_{D_{1}}^{k}\right), \end{array}$$

(28) $$\begin{array}{cc} B^{(2),k} & =G_{k}^{\left(2\right)}\left(Y_{k}^{\left(2\right)},S_{D_{2}}^{k}\right), \end{array}$$

(29) $$\begin{array}{cc} \left(B^{(1),k},B^{(2),k}\right) & =G_{k}^{\left(0\right)}\left(Y_{k}^{\left(0\right)},S_{D_{1}}^{k},S_{D_{2}}^{k}\right). \end{array}$$

If the decoders are invertible, then for each side decoder, knowledge of the side information and the output, e.g., $\left(Y_{k}^{\left(1\right)},S_{D_{1}}^{k}\right)$, is equivalent to knowledge of the side information and the input, $\left(B^{(1),k},S_{D_{1}}^{k}\right)$ [5]. For the single description case, it is shown in [5] that without loss of generality, we can restrict our attention to invertible decoders. Furthermore, when minimizing the average data-rate in a causal source coding scheme, it is optimal to minimize the average data-rate by focusing on schemes with invertible decoders [5].

The following results are used to prove the first main result of this section and are a generalization of ([5] Lemma 4.2) to the MD scenario.

**Lemma** **1** (Feedback Markov Chains)**.** *Consider an MD source coding scheme inside a feedback loop as shown in Figure 2. If Assumption 1 applies and if the decoders are invertible when given the side information, then the Markov chain:*

(30) $$X^{k}{|}_{\phi_{1}}-\left(B_{k}^{\left(1\right)},B_{k}^{\left(2\right)}\right){|}_{\phi_{1}}-\left(Y_{k}^{\left(1\right)},Y_{k}^{\left(2\right)}\right){|}_{\phi_{1}},\;k\in N,$$

*holds, with* $\phi_{1}=\left(B^{(1),k-1},B^{(2),k-1},S_{D_{1}}^{k},S_{D_{2}}^{k}\right)$*.*

*Furthermore, let* $\phi_{2}=\left(B^{(1),k-1},S_{D_{1}}^{k}\right)$ *then:*

(31) $$Y_{k}^{\left(2\right)}{|}_{\phi_{2}}-B_{k}^{\left(1\right)}{|}_{\phi_{2}}-Y_{k}^{\left(1\right)}{|}_{\phi_{2}},\;k\in N,$$

*also holds.*

*Additionally, for* $\phi_{3}=\left(B^{(2),k-1},S_{D_{2}}^{k}\right)$ *:*

(32) $$\begin{array}{c} Y_{k}^{\left(1\right)}{|}_{\phi_{3}}-B_{k}^{\left(2\right)}{|}_{\phi_{3}}-Y_{k}^{\left(2\right)}{|}_{\phi_{3}},\;k\in N, \end{array}$$

*holds.*

*Finally, if the decoder side information is mutually independent, i.e.,* $\left\{S_{D_{1}}\right\}⫫\left\{S_{D_{2}}\right\}$ *, the Markov chains:*

(33) $$\begin{array}{c} Y^{(2),k-1}-Y^{(1),k-1}-S_{D_{1}}^{k},\;k\in N, \end{array}$$

(34) $$\begin{array}{c} Y^{(1),k}-Y^{(2),k-1}-S_{D_{2}}^{k},\;k\in N, \end{array}$$

*hold.*

**Proof.**  The Markov chain in Equation (30) follows, since $Y_{k}^{\left(1\right)},\;Y_{k}^{\left(2\right)}$ depend deterministically upon $\left(B^{(1),k},B^{(2),k},S_{D_{1}}^{k},S_{D_{2}}^{k}\right)$. Similarly, Equation (31) holds, since $Y_{k}^{\left(1\right)}$ depends deterministically upon $\left(B^{(1),k},S_{D_{1}}^{k}\right)$. The Markov chain in Equation (32) follows analogously.

By the system equations, we have that:

(35) $$\begin{array}{cc} \left(B_{1}^{\left(1\right)},B_{1}^{\left(2\right)}\right) & =E_{1}\left(X_{1},\emptyset ,\emptyset ,S_{E,1}\right) \end{array}$$

(36) $$\begin{array}{cc} Y_{1}^{\left(1\right)} & =D_{1}^{\left(1\right)}\left(B_{1}^{\left(1\right)},S_{D_{1},1}\right) \end{array}$$

(37) $$\begin{array}{cc} Y_{1}^{\left(2\right)} & =D_{1}^{\left(2\right)}\left(B_{1}^{\left(2\right)},S_{D_{2},1}\right). \end{array}$$

Since $S_{D_{1},1}⫫S_{D_{2},1}$, it follows that $Y_{1}^{\left(2\right)}⫫S_{D_{1},1}$. Furthermore, since $S_{D_{1},2}⫫S_{D_{2},1}$ then $Y_{1}^{\left(2\right)}⫫S_{D_{1},2}$. Hence, Equation (33) holds in the initial step. Now, in the next time step:

(38) $$\begin{array}{cc} \left(B_{2}^{\left(1\right)},B_{2}^{\left(2\right)}\right) & =E_{2}\left(X^{2},Y_{1}^{\left(1\right)},Y_{1}^{\left(2\right)},S_{E,2}\right) \end{array}$$

(39) $$\begin{array}{cc} \; & =E_{2}\left(X^{2},D_{1}^{\left(1\right)}\left(B_{1}^{\left(1\right)},S_{D_{1},1}\right),Y_{1}^{\left(2\right)},S_{E,2}\right) \end{array}$$

(40) $$\begin{array}{cc} Y_{2}^{\left(1\right)} & =D_{2}^{\left(1\right)}\left(B_{2}^{\left(1\right)},S_{D_{1},2}\right) \end{array}$$

(41) $$\begin{array}{cc} Y_{2}^{\left(2\right)} & =D_{2}^{\left(2\right)}\left(B_{2}^{\left(2\right)},S_{D_{2},2}\right), \end{array}$$

where we see that $Y_{2}^{\left(2\right)}$ depends on $S_{D_{1},1}$ only through $Y_{1}^{\left(1\right)}$. Thus:

(42) $$Y_{2}^{\left(2\right)}-Y_{1}^{\left(1\right)}-S_{D_{1},1}.$$

By the same arguments as before, we have for the second time step $Y_{2}^{\left(2\right)}⫫S_{D_{1},2}$ and $Y_{2}^{\left(2\right)}⫫S_{D_{1},3}$. By the causality of the system components, it follows that $Y^{(2),k-1}$ only depend on $S_{D_{1}}^{k-1}$ through $Y^{(1),k-1}$, and by the independence of the side information, $Y^{(2),k-1}⫫S_{D_{1},k}$; thus. we get Equation (33).

For Equation (34), since $S_{D_{1},1}⫫S_{D_{2},1}$, then $Y_{1}^{\left(1\right)}⫫S_{D_{2},1}$, and the Markov chain holds in the initial step. For the next step, since $Y_{2}^{\left(1\right)}$ depends on $S_{D_{2},1}$ only through $Y_{1}^{\left(2\right)}$, the Markov chain holds. Therefore, by the causality of the system components, $Y_{k}^{\left(1\right)}$ only depends on $S_{D_{2}}^{k-1}$ through $Y^{(2),k-1}$, and because $S_{D_{1},k}⫫S_{D_{2},k}$, it follows that $Y_{k}^{\left(1\right)}⫫S_{D_{2},k}$. Therefore, Equation (34) holds. □

We note that requiring the side information to be mutually independent is not a hard assumption. For example, it is straightforward to generate independent dither signals for two quantizers. A short perspective on removing this assumption is given in Section 6.

We define the mutual information rate between two random processes next.

**Definition** **6** (Mutual information rate ([33] Equation (7.3.9)))**.** *The mutual information rate between two random processes* $\left\{X_{k}\right\}$ *and* $\left\{Y_{k}\right\}$ *is defined as:*

(43) $$\bar{I}\left(X;Y\right)≜\lim_{n\to \infty }\frac{1}{n}I\left(X^{n};Y^{n}\right).$$

We are now ready to state our first main result.

**Theorem** **1** (Lower bound on sum-rate)**.** *Consider a ZDMD source coding problem with feedback (Problem 1), as seen in Figure 2. If Assumption 1 holds, the decoders are invertible, and the decoder side information is mutually independent, then:*

(44) $$R_{1}+R_{2}\geq \bar{I}\left(X\to Y^{\left(1\right)},Y^{\left(2\right)}\right)+\bar{I}\left(Y^{\left(1\right)};Y^{\left(2\right)}\right).$$

The proof of Theorem 1 can be found in Appendix A.

Theorem 1 shows that when imposing zero-delay constraints on MD coding with feedback, the directed information rate from the source to the central reconstruction together with the mutual information rate between the side reconstructions serve as a lower bound on the associated average data sum-rate, thus relating the operational ZDMD rates to the information-theoretic quantities of directed and mutual information rate.

To the best of the authors’ knowledge, Theorem 1 provides a novel characterization between the relationship of the operational sum-rate and directed and mutual information rates, for a ZDMD coding problem with feedback. This result extends on the novel single-description bound in [5] and the MD results of [11].

In relation to the El-Gamal and Cover region [11], our result shows that the first term in the bound on the ZDMD sum-rate, i.e., the no excess sum-rate, is given by the directed information rate from the source to the side descriptions—that is, only the causally conveyed information, as would be expected for ZD coding. The second term is similar to that of El-Gamal and Cover. That is, the excess rate must be spent on communicating the mutual information between the side descriptions to reduce the central distortion.

**Remark** **1.** *The mutual information rate*$\bar{I}\left(Y^{\left(1\right)};Y^{\left(2\right)}\right)$*does not imply a noncausal relationship between*$Y^{\left(1\right)}$*and*$Y^{\left(2\right)}$*, i.e., that*$Y^{\left(1\right)}$*might depend on future values of*$Y^{\left(2\right)}$*. It only implies probabilistic dependence across time [22]. There is feedback between*$Y^{\left(1\right)}$*and*$Y^{\left(2\right)}$*, such that information flows between the two descriptions. However, the information flows in a* causal *manner, i.e., the past values of* $Y^{\left(1\right)}$ *affect the future values of* $Y^{\left(2\right)}$ *and vice versa. This is also apparent from the “delayed” information flow from* $Y^{(2),n-1}$ *to* $Y^{(1),n}$ *in the proof, see Equation* (A7)*. Therefore, the MD code must convey this total information flow between the two descriptions to the central receiver.*

### 3.3. Gaussian Lower Bound For Scalar Gauss-Markov Sources

Before showing Gaussian reproductions minimize the result of Theorem 1, we introduce the following technical assumptions required for our proof.

**Assumption** **2** (Sequential greedy coding)**.** *Consider the ZDMD coding problem in Figure 2. We say that we solve this problem using sequential greedy coding if sequentially for each time step* k∈N*: We minimize the bit-rate such that the MSE distortion constraints* $D_{i}>0,\;i=0,1,2,$ *are satisfied for each* k∈N*.*

*That is, sequentially for each* k∈N *, choose the codewords* $B_{k}^{\left(i\right)},\;i=1,2$ *with minimum codeword lengths* $l_{k}^{\left(i\right)},\;i=1,2$ *such that:*

(45) $$\begin{array}{cc} E\left[{\left(X_{k}-Y_{k}^{\left(0\right)}\right)}^{2}\right] & \leq D_{0} \end{array}$$

(46) $$\begin{array}{cc} E\left[{\left(X_{k}-Y_{k}^{\left(i\right)}\right)}^{2}\right] & \leq D_{i},\;i=1,2. \end{array}$$

Since, in sequential greedy coding, we minimize the bit-rate for each k∈N in the sequential order subject to the distortion constraints, this implies for the information rates in Equation (57) that we minimize the sum:

(47) $$\begin{array}{cc} I\left(X^{n}\to Y^{(1),n},Y^{(2),n}\right)+I\left(Y^{(1),n};Y^{(2),n}\right) & =\sum_{k=1}^{n}[I\left(X^{k};Y_{k}^{\left(1\right)},Y_{k}^{\left(2\right)}|Y^{(1),k-1},Y^{(2),k-1}\right)\\ \; & \;+I\left(Y_{k}^{\left(2\right)};Y_{k}^{\left(1\right)}|Y^{(1),k-1},Y^{(2),k-1}\right)\\ \; & \;+I\left(Y_{k}^{\left(1\right)};Y^{(2),k-1}|Y^{(1),k-1}\right)\\ \; & \;+I\left(Y_{k}^{\left(2\right)};Y^{(1),k-1}|Y^{(2),k-1}\right)], \end{array}$$

by sequentially for each $k\in N^{n}$ selecting the optimal test-channel distribution $P\left(y^{\left(0\right)},y_{k}^{\left(1\right)},y_{k}^{\left(2\right)}|y^{(1),k-1},y^{(2),k-1},y^{(0),k-1},x^{k}\right)$ subject to the MSE distortion constraints:

(48) $$\begin{array}{cc} E\left[{\left(X_{k}-Y_{k}^{\left(0\right)}\right)}^{2}\right] & \leq D_{0}\\ E\left[{\left(X_{k}-Y_{k}^{\left(i\right)}\right)}^{2}\right] & \leq D_{i},\;i=1,2, \end{array}$$

and fixing this distribution for all following $k^{\prime }>k.$

Let ${\tilde{Y}}_{1}^{\left(i\right)},\;i=1,2$ minimize the initial mutual informations for k=1, i.e.:

(49) $$I\left(X_{1};Y_{1}^{\left(1\right)},Y_{1}^{\left(2\right)}\right)+I\left(Y_{1}^{\left(2\right)};Y_{1}^{\left(1\right)}\right)\geq I\left(X_{1};{\tilde{Y}}_{1}^{\left(1\right)},{\tilde{Y}}_{1}^{\left(2\right)}\right)+I\left({\tilde{Y}}_{1}^{\left(2\right)};{\tilde{Y}}_{1}^{\left(1\right)}\right)$$

with equality if $Y_{1}^{\left(i\right)},\;i=1,2$, are distributed as ${\tilde{Y}}_{1}^{\left(i\right)},\;i=1,2$. Then, sequential greedy coding implies $Y_{1}^{\left(i\right)},\;i=1,2$ must be distributed as ${\tilde{Y}}_{1}^{\left(i\right)},\;i=1,2$, for all k>1. Particularly for k=2:

(50) $$\begin{array}{cc} \; & I\left(X^{2};Y_{2}^{\left(1\right)},Y_{2}^{\left(2\right)}|Y_{1}^{\left(1\right)},Y_{1}^{\left(2\right)}\right)+I\left(Y_{2}^{\left(2\right)};Y_{2}^{\left(1\right)}|Y_{1}^{\left(1\right)},Y_{1}^{\left(2\right)}\right)+I\left(Y_{2}^{\left(1\right)};Y_{1}^{\left(2\right)}|Y_{1}^{\left(1\right)}\right)+I\left(Y_{2}^{\left(2\right)};Y_{1}^{\left(1\right)}|Y_{1}^{\left(2\right)}\right)\\ = & I\left(X^{2};Y_{2}^{\left(1\right)},Y_{2}^{\left(2\right)}|{\tilde{Y}}_{1}^{\left(1\right)},{\tilde{Y}}_{1}^{\left(2\right)}\right)+I\left(Y_{2}^{\left(2\right)};Y_{2}^{\left(1\right)}|{\tilde{Y}}_{1}^{\left(1\right)},{\tilde{Y}}_{1}^{\left(2\right)}\right)+I\left(Y_{2}^{\left(1\right)};{\tilde{Y}}_{1}^{\left(2\right)}|{\tilde{Y}}_{1}^{\left(1\right)}\right)+I\left(Y_{2}^{\left(2\right)};{\tilde{Y}}_{1}^{\left(1\right)}|{\tilde{Y}}_{1}^{\left(2\right)}\right), \end{array}$$

where ${\tilde{Y}}_{1}^{\left(i\right)},\;i=1,2$ is inserted on both sides of the conditioning.

The sequential greedy assumption is suitable in a zero-delay source coding perspective, since we must send the optimum description that minimizes the rate while achieving the desired distortion at each time step. We comment on the implications of sequential greedy coding in Section 6.

We also need the following assumption on the minimum MSE (MMSE) predictors.

**Assumption** **3** (Conditional prediction residual independence)**.** *Let* ${\left\{X_{k}\right\}}_{k\in N}$ *be a stationary source process, and let* ${\left\{Y_{k}^{\left(1\right)}\right\}}_{k\in N}$ *and* ${\left\{Y_{k}^{\left(2\right)}\right\}}_{k\in N}$ *be stationary arbitrarily distributed reproduction processes. We say the MMSE reproduction processes have conditional prediction residual independence if the MMSE prediction residuals satisfy for all* k∈N*:*

(51) $$\begin{array}{cccc} Y_{k}^{\left(i\right)}-E\left[Y_{k}^{\left(i\right)}|Y^{(1),k-1},Y^{(2),k-1}\right] & ⫫\left(Y^{(1),k-1},Y^{(2),k-1}\right), & \; & i=1,2, \end{array}$$

(52) $$\begin{array}{cccc} Y_{k}^{\left(i\right)}-E\left[Y_{k}^{\left(i\right)}|Y^{(i),k-1}\right] & ⫫Y^{(i),k-1}, & \; & i=1,2, \end{array}$$

(53) $$\begin{array}{cccc} Y_{k}^{\left(i\right)}-E\left[Y_{k}^{\left(i\right)}|Y^{(j),k-1}\right] & ⫫Y^{(j),k-1}, & \; & i\neq j,\;i,j\in \{1,2\}, \end{array}$$

*that is, the residuals are independent of the conditioning prediction variables.*

For mutual information, the conditional prediction residual independence implies:

(54) $$\begin{array}{cc} \; & I\left(Y_{k}^{\left(1\right)}-E\left[Y_{k}^{\left(1\right)}|Y^{(1),k-1}\right];Y_{k}^{\left(2\right)}-E\left[Y_{k}^{\left(2\right)}|Y^{(1),k-1}\right]|Y^{(1),k-1}\right)\\ = & I\left(Y_{k}^{\left(1\right)}-E\left[Y_{k}^{\left(1\right)}|Y^{(1),k-1}\right];Y_{k}^{\left(2\right)}-E\left[Y_{k}^{\left(2\right)}|Y^{(1),k-1}\right]\right). \end{array}$$

Particularly, if $\{Y_{k}^{\left(i\right)}\},\;i=1,2$ are jointly Gaussian, then the MMSE predictors have conditional prediction residual independence by the orthogonality principle ([34] p. 45). Using these predictors may result in an increased rate, since we limit the amount of possible predictors. That is, by not imposing this condition, we may achieve a smaller distortion for the same rate by minimizing over all possible MMSE predictors.

We are now ready to state our second main result.

**Theorem** **2** (Gaussian lower bound)**.** *Let* ${\left\{X_{k}\right\}}_{k\in N}$ *be a stable stationary scalar Gaussian process* (1) *with nondegenerate MSE distortion constraints,* $D_{i}>0,\;i=0,1,2$*. Then, under the sequential greedy coding condition (Assumption 2), and if the reproduction sequences* $\{Y_{k}^{\left(i\right)}\},\;i=1,2$*, satisfy conditional prediction residual independence (Assumption 3), the following inequality holds:*

(55) $$\begin{array}{c} \bar{I}\left(X\to Y^{\left(1\right)},Y^{\left(2\right)}\right)+\bar{I}\left(Y^{\left(1\right)};Y^{\left(2\right)}\right)\geq \bar{I}\left(X\to Y_{G}^{\left(1\right)},{Y_{G}}^{\left(2\right)}\right)+\bar{I}\left(Y_{G}^{\left(1\right)};Y_{G}^{\left(2\right)}\right), \end{array}$$

*where* $Y_{G}^{\left(i\right)},\;i=1,2$ *are jointly Gaussian random variables with first and second moments equal to those of* $Y^{\left(i\right)},\;i=1,2.$

The proof of Theorem 2 can be found in Appendix B.

Theorem 2 shows that for stationary scalar Gaussian sources under sequential greedy coding and MSE distortion constraints, the mutual informations between the source and side reproductions, and the mutual information between the side reproductions are minimized by Gaussian reproductions. This would generally be expected, since this is the case for single description ZD source coding [8].

To the best of the authors’ knowledge, this is a novel result that has not been documented in any publicly available literature. Similar results exist for single-description ZD source coding [8] and for classical MD coding of white Gaussian sources [35].

**Remark** **2.** *The main difficulty in proving Theorem 2, and the reason for the technical assumptions, is to minimize the excess information rate,*$\bar{I}\left(Y^{\left(1\right)};Y^{\left(2\right)}\right)$*, in Equation* (44) *and show the reconstructions,* $Y^{\left(1\right)},\;Y^{\left(2\right)}$*, should be jointly Gaussian when they are jointly Gaussian with the source. We speculate these technical assumptions may be disregarded, since by the results of [8], we have for a Gaussian source process* $\left\{X_{k}\right\}$*:*

(56) $$\bar{I}\left(X\to Y^{\left(1\right)},Y^{\left(2\right)}\right)\geq \bar{I}\left(X\to Y_{G}^{\left(1\right)},Y_{G}^{\left(2\right)}\right),$$

*with equality if* $\left\{Y_{k}^{\left(1\right)},Y_{k}^{\left(2\right)}\right\}$ *are jointly Gaussian with* $\left\{X_{k}\right\}$*. Therefore, it seems reasonable* $Y^{\left(1\right)},Y^{\left(2\right)}$ *should also be jointly Gaussian in the second term on the RHS of Equation* (44)*. However, we have not been able to prove this.*

#### Symmetric Case

Following the result of Theorem 1, we now formally define the information-theoretic symmetric Gaussian ZDMD RDF, $R_{\mathrm{ZD}}^{I}(D_{0},D_{S})$, in terms of the directed and mutual information rate, as a lower bound to $R_{\mathrm{ZD}}^{\mathrm{op}}(D_{0},D_{S})$. Furthermore, we show that Gaussian reproductions minimize the lower bound.

**Definition** **7** (Information-Theoretic Symmetric ZDMD RDF)**.** *The information-theoretic symmetric ZDMD RDF, for the stationary Gaussian source process* $\left\{X_{k}\right\}$*, with MSE distortion constraints,* $D_{0},D_{S}>0$*, is:*

(57) $$\begin{array}{ccc} R_{ZD}^{I}\left(D_{0},D_{S}\right)≜ & \inf\; & \frac{1}{2}\bar{I}\left(X\to Y^{\left(1\right)},Y^{\left(2\right)}\right)+\frac{1}{2}\bar{I}\left(Y^{\left(1\right)};Y^{\left(2\right)}\right),\\ & s.t. & \lim_{n\to \infty }\frac{1}{n}\sum_{k=1}^{n}E\left[{\left(X_{k}-Y_{k}^{\left(0\right)}\right)}^{2}\right]\leq D_{0}\\ & & \lim_{n\to \infty }\frac{1}{n}\sum_{k=1}^{n}E\left[{\left(X_{k}-Y_{k}^{\left(i\right)}\right)}^{2}\right]\leq D_{S},\;i=1,2, \end{array}$$

*where the infimum is of all processes* $\{Y_{k}^{\left(i\right)}\},\;i=0,1,2$ *that satisfy:*

(58) $$X_{k+1}^{\infty }-X^{k}-\left(Y^{(0),k},Y^{(1),k},Y^{(2),k}\right),\;\forall k\in N.$$

The minimization of all processes $\{Y_{k}^{\left(i\right)}\},\;i=0,1,2$ that satisfy the Markov chain in Equation (58) is equivalent to the minimization of all sequences of conditional test-channel distributions $\left\{P\left(y_{k}^{\left(0\right)},y_{k}^{\left(1\right)},y_{k}^{\left(2\right)}|y^{(0),k-1},y^{(1),k-1},y^{(2),k-1},x^{k}\right):k\in N\right\}$.

For Gaussian reproductions, we have the following optimization problem.

**Problem** **3** (Gaussian Information-Theoretic Symmetric ZDMD RDF)**.** *For a stationary Gaussian source* $\left\{X_{k}\right\}$ *with MSE distortion constraints,* $D_{S}\geq D_{0}>0$*, the Gaussian information-theoretic symmetric ZDMD RDF is:*

(59) $$\begin{array}{ccc} R_{ZD,GM}^{I}\left(D_{0},D_{S}\right)≜ & \inf\; & \frac{1}{2}\bar{I}\left(X\to Y^{\left(1\right)},Y^{\left(2\right)}\right)+\frac{1}{2}\bar{I}\left(Y^{\left(1\right)};Y^{\left(2\right)}\right),\\ & s.t. & \lim_{n\to \infty }\frac{1}{n}\sum_{k=1}^{n}E\left[{\left(X_{k}-Y_{k}^{\left(0\right)}\right)}^{2}\right]\leq D_{0}\\ & & \lim_{n\to \infty }\frac{1}{n}\sum_{k=1}^{n}E\left[{\left(X_{k}-Y_{k}^{\left(i\right)}\right)}^{2}\right]\leq D_{S},\;i=1,2, \end{array}$$

*where the infimum is over all Gaussian processes* $\{Y_{k}^{\left(i\right)}\},\;i=0,1,2$*, that satisfy:*

(60) $$X_{k+1}^{\infty }-X^{k}-\left(Y^{(0),k},Y^{(1),k},Y^{(2),k}\right),\;\forall k\in N.$$

This minimization is equivalent to the minimization of all sequences of Gaussian conditional test-channel distributions

$$\left\{P^{GP}\left(y_{k}^{\left(0\right)},y_{k}^{\left(1\right)},y_{k}^{\left(2\right)}|y^{(0),k-1},y^{(1),k-1},y^{(2),k-1},x^{k}\right):k\in N\right\}.$$

Finally, by Theorems 1 and 2, we have the following corollary, showing Problem 3 as a lower bound to Problem 2.

**Corollary** **1.** *Let*${\left\{X_{k}\right\}}_{k\in N}$*be a stable stationary scalar Gaussian process* (1)*, with MSE distortion constraints,* $D_{S}\geq D_{0}>0$*. Then, under the sequential greedy coding condition (Assumption 2), and if the reproduction sequences* $\{Y_{k}^{\left(i\right)}\},\;i=1,2$*, satisfy conditional prediction residual independence (Assumption 3), the following inequalities hold:*

(61) $$R_{ZD,GM}^{I}\left(D_{0},D_{S}\right)\leq R_{ZD}^{I}\left(D_{0},D_{S}\right)\leq R_{ZD}^{op}\left(D_{0},D_{S}\right).$$

This shows Gaussian reproduction processes minimize the information-theoretic symmetric ZDMD RDF. With this information-theoretic lower bound on $R_{\mathrm{ZD}}^{\mathrm{op}}(D_{0},D_{S})$, we now derive an optimal test-channel realization scheme that achieves this lower bound.

## 4. Symmetric Test-Channel Realization

In this section, we introduce a feedback realization of the optimal test channel for the Gaussian information-theoretic symmetric ZDMD RDF, $R_{\mathrm{ZD},GM}^{I}\left(D_{0},D_{S}\right)$. This test channel is based on the ZDMD coding problem with feedback in Figure 2 and the feedback realization scheme of [8]. Finally, we present a characterization of $R_{\mathrm{ZD},GM}^{I}\left(D_{0},DS\right)$ as the solution to an optimization problem. This provides an achievable lower bound to Problem 2 in a Gaussian coding scheme.

### 4.1. Predictive Coding

**Figure 4:**
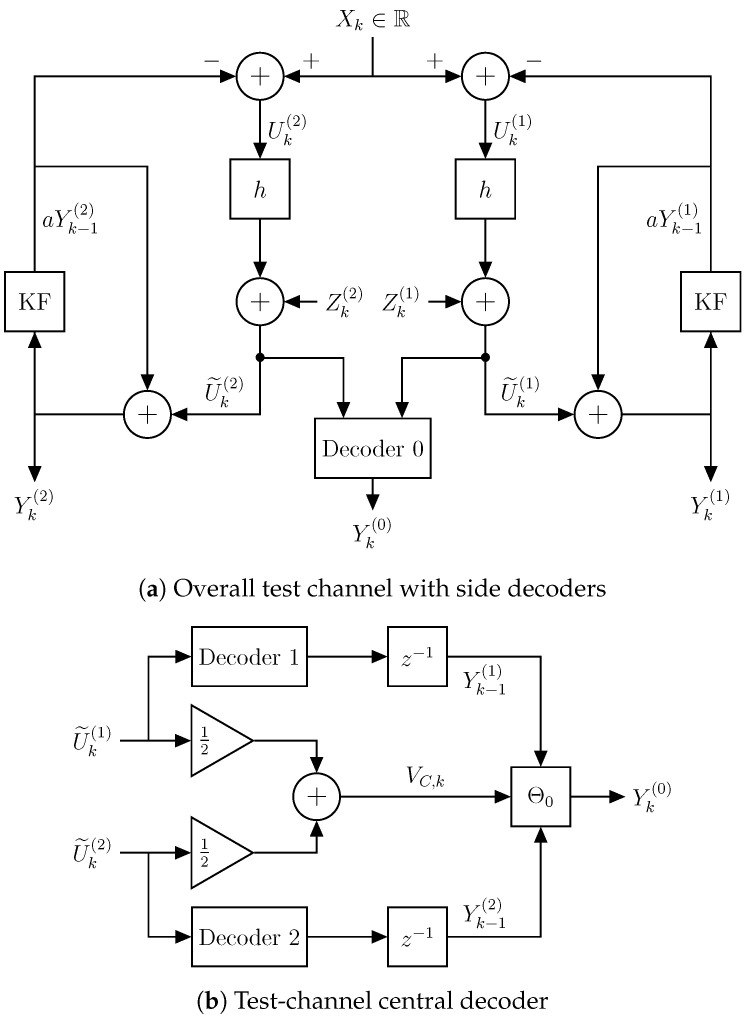
Feedback realization of the optimum test channel for $R_{\mathrm{ZD},GM}^{I}\left(D_{0},DS\right)$.

The feedback realization scheme for the optimum test channel is illustrated in Figure 4. For each side channel, we follow the feedback realization of ([8] Theorem 2). Hence, the reproduction sequence of the optimum test channel is realized by:

(62) $$Y_{k}^{\left(i\right)}=hX_{k}+\left(1-h\right)aY_{k-1}^{\left(i\right)}+Z_{k}^{\left(i\right)},$$

where $Z_{k}^{\left(i\right)}\in R∼N\left(0,\sigma_{Z_{S}}^{2}\right)$:

(63) $$\begin{array}{cc} h & ≜1-\pi_{S}\lambda^{-1}, \end{array}$$

(64) $$\begin{array}{cc} \sigma_{Z_{S}}^{2} & ≜\pi_{S}h, \end{array}$$

(65) $$\begin{array}{cc} \lambda & \;=a^{2}\pi_{S}+\sigma_{W}^{2}. \end{array}$$

Here, λ is the variance of the side error processes:

(66) $$\begin{array}{cc} U_{k}^{\left(i\right)} & ≜X_{k}-E\left[X_{k}|Y^{(i),k-1}\right],\\ \; & \;=X_{k}-aY_{k-1}^{\left(i\right)},\;i=1,2. \end{array}$$

Furthermore, $\pi_{S}$, is the MSE for the estimation of $X_{k}$ and $U_{k}^{\left(i\right)}$, i.e.,:

(67) $$\begin{array}{c} \pi_{S}≜E\left[{\left(X_{k}-Y_{k}^{\left(i\right)}\right)}^{2}\right]=E\left[{\left(U_{k}^{\left(i\right)}-{\tilde{U}}_{k}^{\left(i\right)}\right)}^{2}\right],\;i=1,2, \end{array}$$

where ${\tilde{U}}_{k}^{\left(i\right)}$ are the innovation processes:

(68) $$\begin{array}{cc} {\tilde{U}}_{k}^{\left(i\right)} & ≜Y_{k}^{\left(i\right)}-E\left[Y_{k}^{\left(i\right)}|Y^{(i),k-1}\right] \end{array}$$

(69) $$\begin{array}{cc} \; & \;=hU_{k}^{\left(i\right)}+Z_{k}^{\left(i\right)},\;i=1,2, \end{array}$$

with variance:

(70) $$\begin{array}{cc} \sigma_{\tilde{U}}^{2} & =h^{2}\lambda +\pi_{S}h. \end{array}$$

The innovation process, ${\tilde{U}}_{k}^{\left(i\right)}\;i=1,2,$ can be viewed as the *i*th side decoder estimate of $U_{k}^{\left(i\right)}$.

Finally, we have that:

$$\begin{array}{cc} Z_{k}^{\left(1\right)} & ⫫Z_{l}^{\left(2\right)}\;\forall k\neq \;l\\ Z_{k}^{\left(i\right)} & ⫫Z_{l}^{\left(i\right)}\;\forall k\neq l,\;i=1,2\\ Z_{k}^{\left(i\right)} & ⫫U_{l}^{\left(j\right)}\;\forall k\geq l\;i,\;j\in \left\{1,2\right\}, \end{array}$$

and the joint test-channel noise distribution is:

(71) $$\left[\begin{array}{c} Z_{k}^{\left(1\right)}\\ Z_{k}^{\left(2\right)} \end{array}\right]∼N\left(0,\Sigma_{Z}\right),$$

where:

(72) $$\Sigma_{Z}=\left[\begin{array}{cc} \pi_{S}h & \rho \pi_{S}h\\ \rho \pi_{S}h & \pi_{S}h \end{array}\right].$$

We note that the test channel in Figure 4 differs from the usual MD double-branch test channel of Ozarow [10], since the encoder does not create the two descriptions by adding correlated noises directly to the source, i.e., to the *same* input. Instead, the test channel consists of two branches, each consisting of a differential pulse code modulation (DPCM) scheme, where the correlated noises are added to the two already correlated closed-loop prediction error signals.

We also note the clear resemblance between the ZDMD coding problem in Figure 2 and the test channel in Figure 4a. This shows how the general ZDMD coding problem and its lower bound provide a constructive result that is conveniently extended to an optimum test-channel realization.

### 4.2. Central Decoder Design

The ZDMD encoder creates the two descriptions by prescaling and adding correlated noises to the two prediction error processes, $U_{k}^{\left(1\right)},\;U_{k}^{\left(2\right)}$, resulting in the two innovation processes, ${\tilde{U}}_{k}^{\left(1\right)},{\tilde{U}}_{k}^{\left(2\right)}$, as the side decoder estimates of $U_{k}^{\left(1\right)},\;U_{k}^{\left(2\right)}$. For each time step *k*, the central decoder takes the two innovation processes, ${\tilde{U}}_{k}^{\left(i\right)},\;i=1,2$ as input. Since the additive noises are correlated, the central decoder can provide better estimates of $U_{k}^{\left(1\right)},\;U_{k}^{\left(2\right)}$ than either of the side decoders. Using the central decoder estimates of $U_{k}^{\left(1\right)},U_{k}^{\left(2\right)}$, we can provide a better estimate of the source $X_{k}$ than either side decoder. We average the side innovations processes and define the central innovations description:

(73) $$V_{C,k}≜\frac{1}{2}\left({\tilde{U}}_{k}^{\left(1\right)}+{\tilde{U}}_{k}^{\left(2\right)}\right).$$

Before we discuss the central decoder design, the following lemma provides a useful list of covariances between the signals in the feedback coding scheme of Figure 4, which can be readily verified [27].

**Lemma** **2** (Covariances)**.** *Let* $\left\{X_{k}\right\}$ *be a stable stationary scalar Gauss-Markov process as in Equation* (1) *with stationary variance* $\mathrm{Var}\left[X_{k}\right]=\sigma_{X}^{2}$*. Using the feedback coding scheme of Figure 4, then the following covariances hold:*

(74) $$\begin{array}{cc} \Sigma_{XY} & ≜\mathrm{Cov}\left[X_{k},Y_{k}^{\left(i\right)}\right]=\frac{h}{1-a^{2}\left(1-h\right)}\sigma_{X}^{2},\;i=1,2, \end{array}$$

(75) $$\begin{array}{cc} \Sigma_{XV_{C}} & ≜\mathrm{Cov}\left[X_{k},V_{C,k}\right]=h\left(\sigma_{X}^{2}-a^{2}\Sigma_{XY}\right), \end{array}$$

(76) $$\begin{array}{cc} \sigma_{Y}^{2} & ≜\mathrm{Var}\left[Y_{k}^{\left(i\right)}\right]=\frac{h^{2}\sigma_{X}^{2}+2a^{2}h\left(1-h\right)\Sigma_{XY}+\sigma_{Z_{S}}^{2}}{1-a^{2}{\left(1-h\right)}^{2}},\;i=1,2, \end{array}$$

(77) $$\begin{array}{cc} \Sigma_{Y^{\left(1\right)}Y^{\left(2\right)}} & ≜\mathrm{Cov}\left[Y_{k}^{\left(1\right)},Y_{k}^{\left(2\right)}\right]=\frac{h^{2}\sigma_{X}^{2}+2a^{2}h\left(1-h\right)\Sigma_{XY}+\Sigma_{Z^{\left(1\right)}Z^{\left(2\right)}}}{1-a^{2}{\left(1-h\right)}^{2}} \end{array}$$

(78) $$\begin{array}{cc} \Sigma_{U^{\left(1\right)}U^{\left(2\right)}} & ≜\mathrm{Cov}\left[U_{k}^{\left(1\right)},U_{k}^{\left(2\right)}\right]=\sigma_{X}^{2}+a^{2}\left(\Sigma_{Y^{\left(1\right)}Y^{\left(2\right)}}-2\Sigma_{XY}\right) \end{array}$$

(79) $$\begin{array}{cc} \Sigma_{UV_{C}} & ≜\mathrm{Cov}\left[U_{k}^{\left(i\right)},V_{C,k}\right]=\frac{1}{2}h\left(\lambda +\Sigma_{U^{\left(1\right)}U^{\left(2\right)}}\right),\;i=1,2 \end{array}$$

(80) $$\begin{array}{cc} \sigma_{V_{C}}^{2} & ≜\mathrm{Var}\left[V_{C,k}\right]=\frac{1}{2}\left(\sigma_{\tilde{U}}^{2}+h^{2}\Sigma_{U^{\left(1\right)}U^{\left(2\right)}}+\Sigma_{Z^{\left(1\right)}Z^{\left(2\right)}}\right). \end{array}$$

The central decoder design is illustrated in Figure 4b. For each time step *k*, the central decoder takes the two innovation processes, ${\tilde{U}}_{k}^{\left(i\right)},\;i=1,2$ as input. These are averaged to create the central description $V_{C,k}$. In the previous time step, local side decoders produced the side reconstructions, $Y_{k-1}^{\left(i\right)},\;i=1,2$, such that the central decoder has $Y_{k-1}^{\left(i\right)}\;i=1,2$ available when producing the central estimate, $Y_{k}^{\left(0\right)}$.

Let $\Omega_{k}={\left[V_{C,k},Y_{k-1}^{\left(1\right)},Y_{k-1}^{\left(2\right)}\right]}^{T}$, then the central MMSE estimate of $X_{k}$ is:

(81) $$Y_{k}^{\left(0\right)}=E\left[X_{k}|\Omega_{k}\right]=\Theta_{0}\Omega_{k},$$

where $\Theta_{0}\in R^{1\times 3}$ is given as:

(82) $$\Theta_{0}≜\Sigma_{X\Omega }\Sigma_{\Omega }^{-1},$$

with:

(83) $$\begin{array}{cc} \Sigma_{X\Omega }≜E\left[X_{k}\Omega_{k}^{T}\right] & =\left[\begin{array}{ccc} \Sigma_{XV_{C}} & a\Sigma_{XY} & a\Sigma_{XY} \end{array}\right], \end{array}$$

(84) $$\begin{array}{cc} \Sigma_{\Omega }≜E\left[\Omega_{k}\Omega_{k}^{T}\right] & =\left[\begin{array}{ccc} \sigma_{V_{C}}^{2} & \frac{1}{2}ha\left(\Sigma_{XY}-\Sigma_{Y^{\left(1\right)}Y^{\left(2\right)}}\right) & \frac{1}{2}ha\left(\Sigma_{XY}-\Sigma_{Y^{\left(1\right)}Y^{\left(2\right)}}\right)\\ \frac{1}{2}ha\left(\Sigma_{XY}-\Sigma_{Y^{\left(1\right)}Y^{\left(2\right)}}\right) & \sigma_{Y}^{2} & \Sigma_{Y^{\left(1\right)}Y^{\left(2\right)}}\\ \frac{1}{2}ha\left(\Sigma_{XY}-\Sigma_{Y^{\left(1\right)}Y^{\left(2\right)}}\right) & \Sigma_{Y^{\left(1\right)}Y^{\left(2\right)}} & \sigma_{Y}^{2} \end{array}\right]. \end{array}$$

The central distortion is then:

(85) $$\pi_{0}≜E\left[{\left(X_{k}-Y_{k}^{\left(0\right)}\right)}^{2}\right]=\sigma_{X}^{2}-\Sigma_{X\Omega }\Sigma_{\Omega }^{-1}\Sigma_{X\Omega }^{T}.$$

### 4.3. Rates

We now determine the achievable sum-rate for the test channel.

Initially, for each time step *k*, we express the mutual information in the definition of $R_{\mathrm{ZD},GM}^{I}\left(D_{0},D_{S}\right)$ in Equation (59) using the differential entropy ([28] Ch. 8):

(86) $$\begin{array}{c} I\left(X^{k};Y_{k}^{\left(1\right)},Y_{k}^{\left(2\right)}|Y^{(1),k-1},Y^{(2),k-1}\right)+I\left(Y_{k}^{\left(2\right)};Y^{(1),k}|Y^{(2),k-1}\right)+I\left(Y_{k}^{\left(1\right)};Y^{(2),k-1}|Y^{(1),k-1}\right)\\ =h\left(Y_{k}^{\left(2\right)}|Y^{(2),k-1}\right)+h\left(Y_{k}^{\left(1\right)}|Y^{(1),k-1}\right)-h\left(Y_{k}^{\left(1\right)},Y_{k}^{\left(2\right)}|Y^{(1),k-1},Y^{(2),k-1},X^{k}\right). \end{array}$$

Comparing the test channel of Figure 4 to the general ZDMD source coding scenario with feedback in Figure 2, we have:

(87) $$\begin{array}{cc} h\left(Y_{k}^{\left(i\right)}|Y^{(i),k-1}\right) & =h\left({\tilde{U}}_{k}^{\left(i\right)}\right)=\frac{1}{2}\log\left(2\pi e\lambda h\right) \end{array}$$

and:

(88) $$\begin{array}{cc} h\left(Y_{k}^{\left(1\right)},Y_{k}^{\left(2\right)}|Y^{(1),k-1},Y^{(2),k-1},X^{k}\right)=h\left(Z_{k}^{\left(1\right)},Z_{k}^{\left(2\right)}\right) & =\frac{1}{2}\log\left(2\pi e|\Sigma_{Z}|\right) \end{array}$$

(89) $$\begin{array}{cc} \; & =\frac{1}{2}\log\left(2\pi e\left(\pi_{S}^{2}h^{2}\left(1-\rho^{2}\right)\right)\right). \end{array}$$

Thus, the achievable symmetric sum-rate is:

(90) $$\begin{array}{cc} R_{1}+R_{2} & =\frac{1}{2}\log\left(2\pi e\lambda h\right)+\frac{1}{2}\log\left(2\pi e\lambda h\right)-\frac{1}{2}\log\left(2\pi e\left(\pi_{S}^{2}h^{2}\left(1-\rho^{2}\right)\right)\right) \end{array}$$

(91) $$\begin{array}{cc} \; & =\log\frac{\lambda }{\pi_{S}}-\frac{1}{2}\log\left(1-\rho^{2}\right). \end{array}$$

### 4.4. Scalar Lower Bound Theorem

Summarizing the above derivations, we present the following characterization of the Gaussian information theoretic symmetric ZDMD RDF.

**Theorem** **3** (Characterization of $R_{\mathrm{ZD},GM}^{I}\left(D_{0},D_{S}\right)$)**.** *Consider the stationary scalar Gauss-Markov process of* (1). *Given nondegenerate MSE distortion constraints,* $\left(D_{S},D_{0}\right)$*, where* $0<D_{0}\leq D_{s}\leq \sigma_{X}^{2}$*, the Gaussian information-theoretic symmetric ZDMD RDF,* $R_{ZD,GM}^{I}\left(D_{0},D_{S}\right)$ *is characterized by the solution to the following optimization problem.*

(92) $$\begin{array}{cc} \underset{\left\{\pi_{S},\rho_{0}\right\}}{minimize} & \frac{1}{2}\log\frac{\lambda }{\pi_{S}}-\frac{1}{4}\log\left(1-\rho_{0}^{2}\right)\\ subject\;to & -1\leq \rho_{0}\leq 0\\ \; & 0\leq \pi_{S}\leq \lambda \\ \; & 0\leq \pi_{i}\leq D_{i},\;i=0,S, \end{array}$$

*where:*

(93) $$\begin{array}{cc} \lambda & =a^{2}\pi_{S}+\sigma_{W}^{2}, \end{array}$$

(94) $$\begin{array}{cc} \pi_{0} & =\sigma_{X}^{2}-\Sigma_{X\Omega }\Sigma_{\Omega }^{-1}\Sigma_{X\Omega }^{T}, \end{array}$$

*and* $\Sigma_{X\Omega }\;,\Sigma_{\Omega }$ *are defined in Equations* (83) *and* (84)*.*

**Remark** **3** (Uniqueness of optimal solution). *We believe that the optimal solution to Equation* (92) *is unique. Firstly, the objective function in Equation* (92) *can be shown to be convex in* $\pi_{S}$ *and* $\rho_{0}$*. Furthermore, the slope of the objective is negative for all* $\pi_{S}>0$ *and* $-1<\rho_{0}\leq 0$*. Thus, it decreases monotonically towards a minimum. Additionally, for nondegenerate distortions, there should be equality in the distortions bounds, and since every* $\rho_{0}$ *indicates a certain trade-off point on the dominant face of the rate-distortion region, the minimum should be unique for every fixed* $\rho_{0}$*. Hence, we conjecture the minimum to be unique. However, we have not yet been able to finally prove the uniqueness of the optimal solution to Equation* (92)*.*

This completes the theoretical work on the lower bound to Problem 2, as the solution to Equation (92). Thus, for stationary scalar Gaussian sources in a Gaussian coding scheme, i.e., a source code that achieves the correctly distributed Gaussian noise, we have determined an achievable lower bound to $R_{\mathrm{ZD}}^{\mathrm{op}}(D_{0},D_{S})$, characterized by the (unique) solution to an optimization problem.

We now compare this theoretical lower bound to an operational achievable performance.

## 5. Simulation Study

In this section, we perform two simulation studies to validate our theoretical framework in Section 4 in relation to an operational quantization scheme.

### 5.1. Simple Quantization Scheme

**Figure 5:**
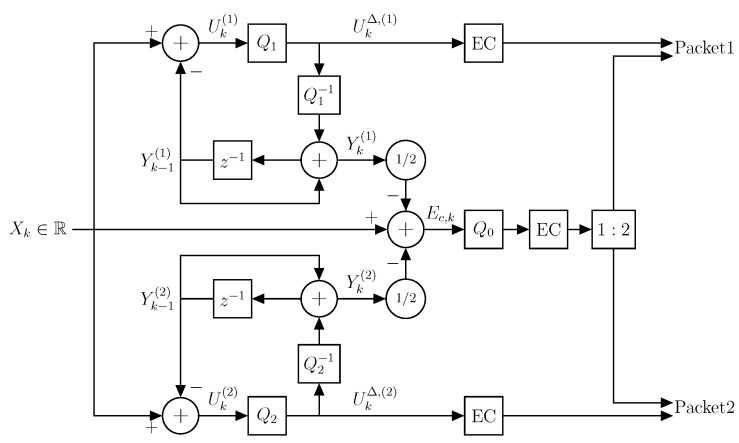
The two-stage staggered differential pulse code modulation (DPCM) quantization scheme. The two first-stage quantizers $Q_{1}$ and $Q_{2}$ are staggered identical uniform quantizers. Here, EC denotes lossless (entropy) encoders. The binary description packets are formed by entropy coding each side quantizer output and splitting the entropy coded second stage quantizer output in two.

In general, test channels provide a basis for the design of practical coding schemes by replacing the additive test-channel noises with quantizers producing quantization noise distributed similar to the test-channel noises. However, it is a nontrivial task to produce quantization noise with high negative correlation in practice [36]. There are some schemes that are able to achieve correlation that tends towards −1 [36], e.g., [37,38,39,40]. These schemes and many other MD coding schemes in general produce two descriptions with the desired correlation by direct operations on the source signal. However, our ZDMD test channel forms two descriptions from two correlated signals. Therefore, many existing schemes are not directly applicable to our test channel. This is somewhat expected since ZDMD coding is mostly an unexplored problem until now. Fortunately, the scheme of [41] illustrated in Figure 5 aligns well with our test channel, since it performs staggered quantization of two prediction error processes and uses a refinement layer for further central distortion gain. The main idea is to use two DPCM encoders with staggered quantizers, $Q_{1}$ and $Q_{2}$, in a base layer and a third second-stage refinement quantizer $Q_{0}$. For a detailed explanation of the derivation and design of this scheme, we refer to [27,41].

### 5.2. Experiments

In all simulations, we consider stationary scalar Gauss-Markov sources of the Form (1). All simulations are conducted by fixing the average rate per description, *R*, given as:

(95) $$R=R_{S}+\frac{R_{0}}{2},$$

where $R_{S}$ is the rate of the first-stage quantizers, $Q_{1},\;Q_{2}$, and $R_{0}$ is the rate of the second-stage (central) quantizer, $Q_{0}$. Then, for each rate-pair, $R_{S},\;R_{0}$, satisfying the rate constraint *R*, the practical quantizer step sizes are determined according to the high-rate approximations:

(96) $$\begin{array}{cc} R_{S} & =H\left(U^{\Delta_{S},\left(i\right)}\right)\approx h\left(U^{\left(i\right)}\right)-\log\Delta_{S}, \end{array}$$

(97) $$\begin{array}{cc} R_{0} & =H\left(E_{C}^{\Delta_{0}}\right)\approx h\left(E_{C}\right)-\log\Delta_{0}, \end{array}$$

where $U^{\Delta_{S},\left(i\right)}$ is the quantized version of $U^{\left(i\right)}$, $E_{C}^{\Delta_{0}}$ is the quantized version of $E_{C}$, and the approximations follow from ([28] Theo. 8.3.1). The step sizes are determined such that the operational rate per description, $R_{op}$, is approximately equal to the constraint, i.e., $R_{op}\approx R$. Further details on choosing the step size is found in [27]. From simulations we have seen, there is an approximate rate-loss of 0.1 bits/sample/description due to the approximation of step sizes in Equations (96) and (97). We have accounted for this when choosing the step sizes, such that $R_{op}$ approximates *R* with greater accuracy. For lower rates, this difference is higher; hence, we consider only the high-rate scenario.

We consider *N* source samples that are independently coded and decoded by the operational quantization scheme, and *M* Monte Carlo simulations for each rate-pair $R_{0},\;R_{S}$. The numerical distortions are obtained by:

(98) $$\begin{array}{cc} {\hat{D}}_{i} & =\frac{1}{N}\sum_{i=k}^{N}{\left(X_{k}-Y_{k}^{\left(i\right)}\right)}^{2},\;\;i=0,1,2, \end{array}$$

(99) $$\begin{array}{cc} {\hat{D}}_{S} & =\frac{{\hat{D}}_{1}+{\hat{D}}_{2}}{2}, \end{array}$$

where $Y_{k}^{\left(i\right)}\;i=0,1,2$ are the reconstructions for the *k*th input sample $X_{k}$. The operational coding rates are determined by the discrete entropies:

(100) $$\begin{array}{cc} {\hat{R}}_{i} & =H\left({\left\{U_{k}^{\Delta_{S},\left(i\right)}\right\}}_{k=1}^{N}\right),\;i=1,2, \end{array}$$

(101) $$\begin{array}{cc} {\hat{R}}_{S} & =\frac{{\hat{R}}_{1}+{\hat{R}}_{2}}{2}, \end{array}$$

(102) $$\begin{array}{cc} {\hat{R}}_{0} & =H\left({\left\{E_{C,k}^{\Delta_{0}}\right\}}_{k=1}^{N}\right), \end{array}$$

where the entropies are determined from the empirical probabilities, which are obtained based on the histograms of ${\left\{U_{k}^{\Delta_{S},\left(i\right)}\right\}}_{k=1}^{N},\;i=1,2$ and ${\left\{E_{C,k}^{\Delta_{0}}\right\}}_{k=1}^{N}$.

The theoretical distortion limits for a given rate *R* are determined by fixing the objective function value in Equation (92), and determining the corresponding $\rho_{0}$ and central distortion $\pi_{0}$ for a grid of side distortions, $\pi_{S}$.

#### 5.2.1. Distortion Trade-Off at Fixed Rate

We consider the trade-off between the side and central distortions, $D_{S}$, $D_{0}$ for a fixed rate per description, R=5 bits/sample. We compare the theoretical lower bound on the distortions to the operational distortions obtained using the practical quantization scheme. The source and simulation parameters are listed in Table 1.

**Figure 6:**
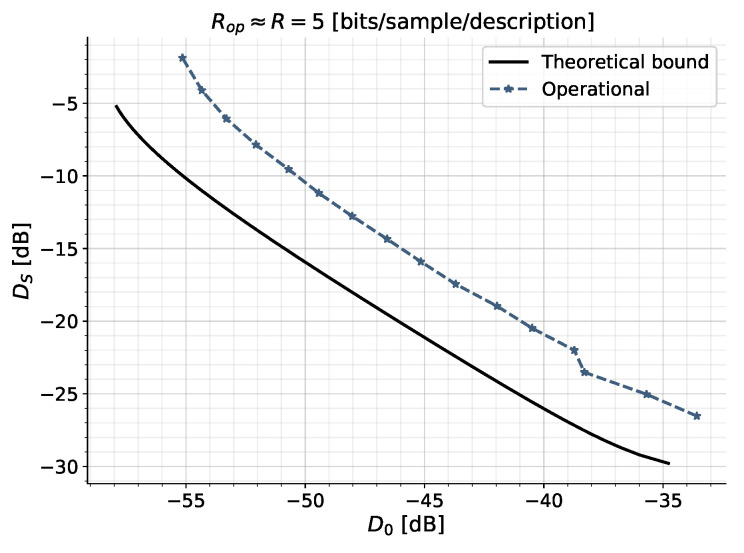
The central distortion, $D_{0}$, versus side distortion, $D_{S}$ for ZDMD coding of a Gauss-Markov source (1) with a=0.9 and unit variance at R=5 bits/sample/description. Simulation parameters in Table 1.

The resulting theoretical and operational distortion curves are shown in Figure 6. The figure shows the theoretical lower bound (black curve) on the achievable distortion region, and the operational achievable distortion pairs (dashed blue curve), for the fixed rate per description R=5 bits/sample. The operational curve lies approximately 5 dB above the theoretical lower bound. Both curves show that if we decrease the central distortion, we must increase the side distortion, and vice-versa, if we want to maintain the same rate *R*. Hence, we are able to trade off between the side- and central distortion by varying the bit allocation in the first- and second stage quantizers.

The 5 dB distortion loss corresponds to a total rate loss of approximately 0.83 bits/sample, for the sum-rate, or equivalently 0.415 bits/sample/description. Some of this loss can be attributed to the space-filling loss of the uniform quantizers, which is approximately 1.5, or 0.254 bits/sample per quantizer. Thus, the refinement scheme suffers from the space-filling loss of three quantizers [42]. Furthermore, there is a loss due to the non-optimal linear predictors; however, this loss is minimal in the high-rate scenario [41].

The sudden bend in the operational curve can be attributed to a possible alphabet change, i.e., for certain rates and, hence, quantization bin sizes, the quantized signals have an increased alphabet size, due to smaller bin sizes.

#### 5.2.2. Distortion versus Distortion-Ratio for Multiple Fixed Rates

**Figure 7:**
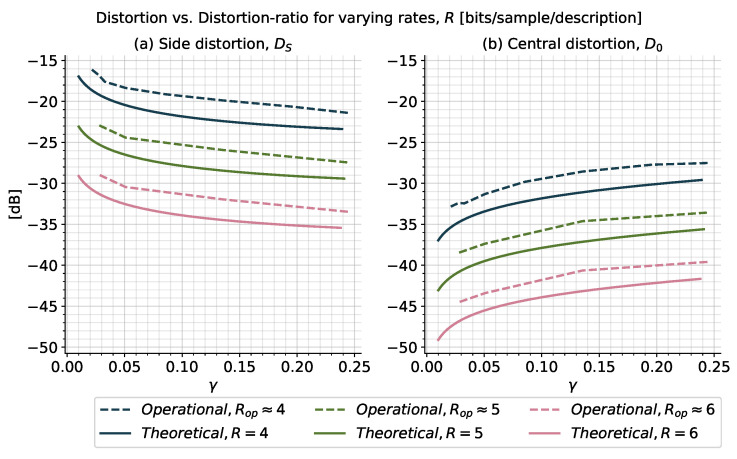
(**a**) Side distortion, $D_{S}$, and (**b**) central distortion, $D_{0}$, versus distortion ratio $\gamma =D_{0}/D_{S}$ for ZDMD coding of a Gauss-Markov source (1) with a=0.9 and unit variance at R∈{4,5,6} bits/sample/description. Simulation parameters in Table 2.

We next consider how the side- and central distortions, $D_{0},D_{S}$, vary with the distortion ratio $\gamma ≜D_{0}/D_{S}$ for different fixed rates *R*. Using the previously described procedure for the fixed rates R∈{4,5,6} bits/sample/description, we obtained the distortion curves in Figure 7; the simulation parameters are listed in Table 2. Figure 7a shows the side distortion, $D_{S}$, in relation to the distortion ratio, γ, for varying rates. Similarly, Figure 7b shows the central distortion, $D_{0}$, in relation to the distortion ratio, γ, for the same rates. In both figures, dashed curves indicate operational distortions and ratios, and solid curves indicate theoretical bounds.

For any particular rate and distortion ratio in Figure 7, the central distortion, $D_{0}$, is always lower than the side distortion, $D_{S}$. Further, as the rate per description increases, both distortions decrease for all distortion ratios. Lower ratios imply lower central distortion, $D_{0}$, at the cost of a higher side distortion $D_{S}$. This was also seen in Figure 6. Figure 7 shows, this trend is independent of the rate. Furthermore, the plots in Figure 7 show that by increasing the rate per description for any fixed ratio, we can increase the performance in both central and side distortion.

It can be shown, at no excess marginal rate, i.e., when $R_{0}=0$, we have that $D_{0}/D_{S}\approx 1/4$ [27], and therefore, the maximum operational distortion ratio is limited to approximately 1/4. Hence, to evaluate higher distortion ratios, we would need to perform non-optimal central reconstructions or decrease the quantizer offsets away from the optimum half bin size.

For a given rate and distortion ratio, the operational curves in Figure 7 are all approximately 2.5 dB above the theoretical bounds, with a slightly better performance at higher rates. This loss can again be attributed to the space-filling loss and non-optimal predictors. We notice that this loss seems to be half of that seen when plotting $D_{S}$ versus $D_{0}$ in Figure 6. However, for a given ratio, there are two curves in Figure 7, one for each of $D_{S}$ and $D_{0}$. Thus, the total distortion loss at a give ratio is 5 dB. Therefore, the apparent splitting of the loss can be attributed to a 2.5 dB loss for each of $D_{S}$ and $D_{0}$ at a given ratio, c.f. [27].

From the rate-distortion performances in Figure 6 and Figure 7, we see for the high-rate scenario that the simple quantization scheme is able to achieve a performance close to the theoretical ZDMD lower bounds derived in the previous sections. Hence, we are able to operate along the theoretical bounds for ZDMD coding of stationary scalar Gaussian sources using simple techniques. Particularly, we are able to trade off both rates and distortions. The simulation results also provide an indication of an upper bound on the optimal operational performance limits of ZDMD coding of stationary scalar Gauss-Markov sources.

## 6. Discussion

We now discuss some important aspects of our derivations and simulation results. Particularly, we focus on the assumptions made in the information-theoretic lower bound derivation, and how the test channel generalizes to an operational quantization scheme. Finally, we consider extension of our results to vector Gauss-Markov sources.

### 6.1. Theoretical Lower Bound

In order to derive an information-theoretic lower bound on the symmetric ZDMD RDF for scalar stationary Gaussian sources in Theorem 2, we have made some technical assumptions.

The main assumption was the use of sequential greedy coding (Assumption 2). This implies that at each time step, we must encode a source sample such that the rates are minimized and the distortion constraints are achieved. However, this might lead to an increased rate, since we must achieve the desired distortion performance in each time step and not just in the asymptotic average. Hence, for some source samples, excess bits might have to be spent to ensure the distortion constraints are achieved. The reason for this technical assumption is its implication from an information-theoretic or probabilistic point of view. That is, the test-channel distribution of a particular reconstruction given the current and past inputs should remain unchanged once it has been selected. It seems plausible that sequential greedy coding provides the same ZDMD information rates as jointly selecting the optimal test-channel distribution over all time steps. Since, from a ZD perspective, all source samples must be encoded and transmitted immediately without delay, their respective reconstruction distributions are thus selected only once. However, this remains an open problem for future research.

To derive the information-theoretic lower bound on the sum-rate, we assume the decoder side information is mutually independent. This assumption ensures the side-decoder reproduction, $Y_{k}^{\left(1\right)}$, is independent of the side information belonging to the other decoder, $S_{D_{2}}^{k}$, when the previous reproductions, $Y^{(2),k-1}$ are given, and vice versa for reproduction $Y_{k}^{\left(2\right)}$. Therefore, if using dependent or common side information, the results of Section 3 warrant further investigation, although, for common side information, it seems reasonable that the bounds should remain widely unchanged. In [43], an achievable region is derived for MD coding without feedback and with common side information, in the classic distributed information-theoretic sense. The bounds of [43] are similar to those of El-Gamal and Cover [11] with an added dependency upon the unknown side information in the involved mutual informations. Hence, these results could provide a basis for extending the results of Section 3 to the case of unknown or dependent side information.

### 6.2. Difficulties with the Vector Case

We note that Problem 2 and our first main result of Theorem 1 also hold for stationary vector sources. Similarly, the definition of the (Gaussian) information-theoretic symmetric ZDMD RDF is easily extended to the vector case.

The main concern is that of extending Theorem 2 to the vector case, i.e., showing Gaussian reproductions minimize the information theoretic lower bound on the sum-rate for stationary Gaussian vector sources. In [35], the scalar result of Ozarow is extended to IID Gaussian vector processes and shows the natural Gaussian multiple description scheme is optimal in achieving the lower bound on the sum-rate for matrix covariance constraints. In [14], it is shown how the Gaussian description scheme is also optimal under MSE distortion constraints. In the sense of zero delay, the results of [8,17] show that for Gauss-Markov source processes, the jointly Gaussian reproduction process minimizes the information-theoretic lower bound. Therefore, based on these results, we conjecture the result of Theorem 2 may be extended to Gaussian vector processes. To this end, we note that the proof of Theorem 2 relies on the tightness of Ozarow’s lower bound for stationary scalar Gaussian processes. This reliance on scalar sources may be disregarded if it can be shown that:

(103) $$\bar{I}\left(Y^{\left(1\right)};Y^{\left(2\right)}\right)\geq \bar{I}\left(Y_{G}^{\left(1\right)};Y_{G}^{\left(2\right)}\right),$$

with equality if $Y^{\left(1\right)},Y^{\left(2\right)}$ are jointly Gaussian. For some initial results in this regard, see the extended proof of Theorem 2 in ([27] App. E).

If Theorem 2 can be extended to the vector case, it remains to derive an optimum test-channel realization scheme. Early work by the authors indicates that the test channel in Section 4 may be generalized to the vector case in a similar manner to that of [8]. In the stationary case, the covariances in Lemma 2 may be extended to the vector case in the form of Ricatti matrix equations, where explicit solutions may be obtained using the techniques of ([44] Section 5). However, the main difficulty is that of determining the proper correlation between Gaussian test-channel noise vectors. Particularly, due to the structure of the noise covariance matrix, it is difficult to derive expressions for the determinant of $\Sigma_{Z}$ such that a more explicit, possibly using semidefinite programming, expression may be formulated for $R_{\mathrm{ZD},GM}^{I}\left(D_{0},D_{S}\right)$. For spatially uncorrelated vector sources, the extension is fairly straightforward, since it can be reasonably assumed that the noise cross-covariance matrix $\Sigma_{Z^{\left(1\right)}Z^{\left(2\right)}}$ should be diagonal along with $\Sigma_{Z_{S}}$. Since the dimensions are independent, the scalar solution can be applied to each dimension, and the total rates and distortions are given as sums across the scalar solutions for each dimension.

## 7. Conclusions

In this work, we studied the ZDMD source coding problem where the MD encoder and decoders are required to be causal and of zero delay. Furthermore, the encoder receives perfect decoder feedback, and side information is available to both encoder and decoders. Using this constructive system, we showed that the average data sum-rate is lower bounded by the sum of the directed information rate from the source, *X*, to the side descriptions, $Y^{\left(1\right)},Y^{\left(2\right)}$, and the mutual information rate between the side descriptions, thus providing a novel relation between information theory and the operational ZDMD coding rates.

For scalar stationary Gaussian sources with MSE distortion constraints subject to the technical assumptions of sequential greedy coding and conditional residual independence, we showed this information-theoretic lower bound is minimized by Gaussian reproductions, i.e., the optimum test-channel distributions are Gaussian. This bound provides an information-theoretic lower bound to the operational symmetric ZDMD RDF, $R_{\mathrm{ZD}}^{\mathrm{op}}(D_{0},D_{S})$.

We showed the optimum test channel of the Gaussian information-theoretic lower bound is determined by a feedback realization scheme utilizing predictive coding and correlated Gaussian noises. This shows that the information-theoretic lower bound for first-order stationary scalar Gauss-Markov sources is achievable in a Gaussian coding scheme. Additionally, the optimum Gaussian test-channel distribution is characterized by the solution to an optimization problem.

We have not yet been able to extend the test channel into an operational quantization scheme that allows for an exact upper bound on the optimum operational performance limits.

Operational achievable results are determined for the high-rate scenario by utilizing the simple quantization scheme of [41], resembling our test channel to some extent. Using this simple quantization scheme, it is possible to achieve operational rates within 0.415 bits/sample/description of the theoretical lower bounds.

## Figures and Tables

**Table 1:** Simulation Parameters for distortion trade-off curve in Figure 6.

Source Parameters	Symbol	Values
Source correlation coefficient	*a*	0.9
Source innovation variance	$\sigma_{W}^{2}$	1
Initial value variance	$\sigma_{X_{1}}^{2}$	$\frac{1}{1-0.9^{2}}$
Simulation parameters	Symbol	Values
Rate per description	*R*	5 bits/sample
Time samples	*N*	500,000
Monte-Carlo simulations	*M*	4

**Table 2:** Simulation parameters for distortion versus distortion ratio curves in Figure 7.

Source Parameters	Symbol	Values
Source correlation coefficient	*a*	0.9
Source innovation variance	$\sigma_{W}^{2}$	1
Initial value variance	$\sigma_{X_{1}}^{2}$	$\frac{1}{1-0.9^{2}}$
Simulation parameters	Symbol	Values
Rate per description	*R*	{4,5,6} bits/sample
Time samples	*N*	500,000
Monte-Carlo simulations	*M*	4

## Abbreviations

The following abbreviations are used in this manuscript:

DPCM	Differential Pulse Code Modulation
DPI	Data Processing Inequality
IID	Independent and Identically Distributed
MD	Multiple Description
MMSE	Minimum Mean-Squared Error
MSE	Mean-Squared Error
RDF	Rate-Distortion Function
ZD	Zero Delay
ZDMD	Zero-Delay Multiple Description

## Appendix A. Proof of Theorem 1

First, since the expected length of a uniquely decodable code is lower bounded by its entropy ([28] Ch. 5), we have that:

(A1) $$\begin{array}{cc} E\left[l_{k}^{\left(i\right)}\right] & \geq H\left(B_{k}^{\left(1\right)}|B^{(i),k-1},S_{D_{i}}^{k}\right),\;i=1,2, \end{array}$$

since $B^{(i),k-1}$ and $S_{D_{i}}^{k}$ are already available at decoder *i* before the reception of $B_{k}^{\left(i\right)}$. Thus:

(A2) $$\begin{array}{cc} E\left[l_{k}^{\left(1\right)}\right]+E\left[l_{k}^{\left(2\right)}\right]\; & \geq H\left(B_{k}^{\left(1\right)}|B^{(1),k-1},S_{D_{1}}^{k}\right)+H\left(B_{k}^{\left(2\right)}|B^{(2),k-1},S_{D_{2}}^{k}\right)\\ \; & \overset{\left(a\right)}{\geq }H\left(B_{k}^{\left(1\right)}|B^{(1),k-1},S_{D_{1}}^{k}\right)+H\left(B_{k}^{\left(2\right)}|B^{(2),k-1},S_{D_{2}}^{k}\right)\\ \; & \;-H\left(B_{k}^{\left(1\right)},B_{k}^{\left(2\right)}|B^{(1),k-1},B^{(2),k-1},X^{k},S_{D_{1}}^{k},S_{D_{2}}^{k}\right)\\ \; & \overset{\left(b\right)}{=}H\left(B_{k}^{\left(1\right)}|B^{(1),k-1},S_{D_{1}}^{k}\right)-H\left(B_{k}^{\left(1\right)},B_{k}^{\left(2\right)}|B^{(1),k-1},B^{(2),k-1},S_{D_{1}}^{k},S_{D_{2}}^{k}\right)\\ \; & \;+H\left(B_{k}^{\left(2\right)}|B^{(2),k-1},S_{D_{2}}^{k}\right)+I\left(X^{k};B_{k}^{\left(1\right)},B_{k}^{\left(2\right)}|B^{(1),k-1},B^{(2),k-1},S_{D_{1}}^{k},S_{D_{2}}^{k}\right)\\ \; & \overset{\left(c\right)}{=}H\left(B_{k}^{\left(1\right)}|B^{(1),k-1},S_{D_{1}}^{k}\right)+H\left(B_{k}^{\left(2\right)}|B^{(2),k-1},S_{D_{2}}^{k}\right)-H\left(B_{k}^{\left(1\right)}|B^{(1),k-1},B^{(2),k-1},S_{D_{1}}^{k},S_{D_{2}}^{k}\right)\\ \; & \;-H\left(B_{k}^{\left(2\right)}|B^{(1),k},B^{(2),k-1},S_{D_{1}}^{k},S_{D_{2}}^{k}\right)+I\left(X^{k};B_{k}^{\left(1\right)},B_{k}^{\left(2\right)}|B^{(1),k-1},B^{(2),k-1},S_{D_{1}}^{k},S_{D_{2}}^{k}\right)\\ \; & \overset{\left(d\right)}{=}I\left(X^{k};B_{k}^{\left(1\right)},B_{k}^{\left(2\right)}|B^{(1),k-1},B^{(2),k-1},S_{D_{1}}^{k},S_{D_{2}}^{k}\right)+I\left(B_{k}^{\left(2\right)};B^{(1),k},S_{D_{1}}^{k}|B^{(2),k-1},S_{D_{2}}^{k}\right)\\ \; & \;+I\left(B_{k}^{\left(1\right)};B^{(2),k-1},S_{D_{2}}^{k}|B^{(1),k-1},S_{D_{1}}^{k}\right), \end{array}$$

where (a) follows from the non-negativity of discrete entropy ([28] Lem. 2.1.1). Step (b) follows from the definition of conditional mutual information ([28] p. 23), (c) by the chain rule for discrete entropy ([28] Theo. 2.5.1), and (d) by the definition of conditional mutual information ([28] p. 23). Consider the first term of step (d) in Equation (A2):

(A3) $$\begin{array}{cc} I\left(X^{k};B_{k}^{\left(1\right)},B_{k}^{\left(2\right)}|B^{(1),k-1},B^{(2),k-1},S_{D_{1}}^{k},S_{D_{2}}^{k}\right)\; & \overset{\left(e1\right)}{=}I\left(X^{k};B_{k}^{\left(1\right)},B_{k}^{\left(2\right)}|Y^{(1),k-1},Y^{(2),k-1},S_{D_{1}}^{k},S_{D_{2}}^{k}\right)\\ \; & \overset{\left(e2\right)}{\geq }I\left(X^{k};Y_{k}^{\left(1\right)},Y_{k}^{\left(2\right)}|Y^{(1),k-1},Y^{(2),k-1},S_{D_{1}}^{k},S_{D_{2}}^{k}\right)\\ \; & \overset{\left(e3\right)}{=}I\left(X^{k};Y^{(1),k},Y^{(2),k},S_{D_{1}}^{k},S_{D_{2}}^{k}\right)-I\left(X^{k};Y^{(1),k-1},Y^{(2),k-1},S_{D_{1}}^{k},S_{D_{2}}^{k}\right)\\ \; & \overset{\left(e4\right)}{\geq }I\left(X^{k};Y^{(1),k},Y^{(2),k}\right)-I\left(X^{k};Y^{(1),k-1},Y^{(2),k-1},S_{D_{1}}^{k},S_{D_{2}}^{k}\right)\\ \; & \overset{\left(e5\right)}{=}I\left(X^{k};Y_{k}^{\left(1\right)},Y_{k}^{\left(2\right)}|Y^{(1),k-1},Y^{(2),k-1}\right)\\ \; & \;-I\left(X^{k};S_{D_{1}}^{k},S_{D_{2}}^{k}|Y^{(1),k-1},Y^{(2),k-1}\right)\\ \; & \overset{\left(e6\right)}{=}I\left(X^{k};Y_{k}^{\left(1\right)},Y_{k}^{\left(2\right)}|Y^{(1),k-1},Y^{(2),k-1}\right), \end{array}$$

where (e1) follows since the decoders are invertible given the side information, (e2) follows from the data processing inequality (DPI) ([28] Section 2.8), the invertible decoders, and Equation (30), (e3) by the chain rule of mutual information ([28] Theo. 2.5.2), (e4) since, by the non-negativity of mutual information ([28] Section 2.6), removing a variable can only decrease the mutual information, (e5) by the chain rule of mutual information, and (e6) since the side information is assumed to be independent of *X*.

For the second term of step (d) in Equation (A2):

(A4) $$\begin{array}{cc} I\left(B_{k}^{\left(2\right)};B^{(1),k},S_{D_{1}}^{k}|B^{(2),k-1},S_{D_{2}}^{k}\right) & \overset{\left(f1\right)}{=}I\left(B_{k}^{\left(2\right)};Y^{(1),k},S_{D_{1}}^{k}|Y^{(2),k-1},S_{D_{2}}^{k}\right)\\ \; & \overset{\left(f2\right)}{\geq }I\left(Y_{k}^{\left(2\right)};Y^{(1),k},S_{D_{1}}^{k}|Y^{(2),k-1},S_{D_{2}}^{k}\right)\\ \; & \overset{\left(f3\right)}{\geq }I\left(Y_{k}^{\left(2\right)};Y^{(1),k}|Y^{(2),k-1},S_{D_{2}}^{k}\right)\\ \; & \overset{\left(f4\right)}{=}I\left(Y_{k}^{\left(2\right)},S_{D_{2}}^{k};Y^{(1),k}|Y^{(2),k-1}\right)-I\left(S_{D_{2}}^{k};Y^{(1),k}|Y^{(2),k-1}\right)\\ \; & \overset{\left(f5\right)}{\geq }I\left(Y_{k}^{\left(2\right)};Y^{(1),k}|Y^{(2),k-1}\right)-I\left(S_{D_{2}}^{k};Y^{(1),k}|Y^{(2),k-1}\right)\\ \; & \overset{\left(f6\right)}{=}I\left(Y_{k}^{\left(2\right)};Y^{(1),k}|Y^{(2),k-1}\right), \end{array}$$

where (f1) follows since the decoders are invertible, and (f2) from the DPI and Equation (32), (f3) since conditional mutual information is non-negative, removing a term on the left side of the conditioning can only decrease the mutual information, (f4) follows from the chain rule of mutual information, (f5) is similar to (f3), and finally, (f6) follows from Equation (34), and the mutual information is zero for independent variables.

For the third term of step (d) in Equation (A2), we have through similar derivations using the Markov chains in Equations (31) and (33):

(A5) $$\begin{array}{c} I\left(B_{k}^{\left(1\right)};B^{(2),k-1},S_{D_{2}}^{k}|B^{(1),k-1},S_{D_{1}}^{k}\right)\geq I\left(Y_{k}^{\left(1\right)};Y^{(2),k-1}|Y^{(1),k-1}\right). \end{array}$$

Then, by Equations (A2)–(A5):

(A6) $$\begin{array}{cc} E\left[l_{k}^{\left(1\right)}\right]+E\left[l_{k}^{\left(2\right)}\right] & \geq I\left(X^{k};Y_{k}^{\left(1\right)},Y_{k}^{\left(2\right)}|Y^{(1),k-1},Y^{(2),k-1}\right)+I\left(Y_{k}^{\left(2\right)};Y^{(1),k}|Y^{(2),k-1}\right)+I\left(Y_{k}^{\left(1\right)};Y^{(2),k-1}|Y^{(1),k-1}\right). \end{array}$$

Summing over *k*, we have, by the definition of directed information (Definition 4):

(A7) $$\begin{array}{cc} \sum_{k=1}^{n}\left(E\left[l_{k}^{\left(1\right)}\right]+E\left[l_{k}^{\left(2\right)}\right]\right) & \geq I\left(X^{n}\to Y^{(1),n},Y^{(2),n}\right)+I\left(Y^{(1),n}\to Y^{(2),n}\right)+I\left(Y^{(2),n-1}\to Y^{(1),n}\right)\\ \; & =I\left(X^{n}\to Y^{(1),n},Y^{(2),n}\right)+I\left(Y^{(1),n}\to Y^{(2),n}\right)+I\left(0*Y^{(2),n-1}\to Y^{(1),n}\right)\\ \; & =I\left(X^{n}\to Y^{(1),n},Y^{(2),n}\right)+I\left(Y^{(1),n};Y^{(2),n}\right), \end{array}$$

where the last equality follows from the conservation of information ([45] Prop. 2) and $0*Y^{(2),n-1}$ denotes the concatenation $0,Y_{1}^{\left(2\right)},\ldots ,Y_{n-1}^{\left(2\right)}$. The lower bound (Equation (44)) now follows by dividing by *n* and taking the limit as n→∞. □

## Appendix B. Proof of Theorem 2

Recall:

(A8) $$\begin{array}{cc} \bar{I}\left(X\to Y^{\left(1\right)},Y^{\left(2\right)}\right)+\bar{I}\left(Y^{\left(1\right)};Y^{\left(2\right)}\right) & =\lim_{n\to \infty }\frac{1}{n}I\left(X^{n}\to Y^{(1),n},Y^{(2),n}\right)+\frac{1}{n}I\left(Y^{(1),n};Y^{(2),n}\right) \end{array}$$

where:

(A9) $$\begin{array}{c} I\left(X^{n}\to Y^{(1),n},Y^{(2),n}\right)=\sum_{k=1}^{n}I\left(X^{k};Y_{k}^{\left(1\right)},Y_{k}^{\left(2\right)}|Y^{(1),k-1},Y^{(2),k-1}\right), \end{array}$$

and:

(A10) $$\begin{array}{cc} I\left(Y^{(1),n};Y^{(2),n}\right) & =I\left(Y^{(1),n}\to Y^{(2),n}\right)+I\left(0*Y^{(2),n-1}\to Y^{(1),n}\right) \end{array}$$

(A11) $$\begin{array}{cc} \; & =I\left(Y^{(1),n}\to Y^{(2),n}\right)+I\left(Y^{(2),n-1}\to Y^{(1),n}\right) \end{array}$$

(A12) $$\begin{array}{cc} \; & =\sum_{k=1}^{n}I\left(Y_{k}^{\left(2\right)};Y^{(1),k}|Y^{(2),k-1}\right)+I\left(Y_{k}^{\left(1\right)};Y^{(2),k-1}|Y^{(1),k-1}\right). \end{array}$$

For each time step k∈N, using the chain rule of mutual information [28], we have that:

(A13) $$\begin{array}{cc} \; & I\left(Y_{k}^{\left(2\right)};Y^{(1),k}|Y^{(2),k-1}\right)+I\left(Y_{k}^{\left(1\right)};Y^{(2),k-1}|Y^{(1),k-1}\right)\\ \; & =I\left(Y_{k}^{\left(2\right)};Y_{k}^{\left(1\right)}|Y^{(1),k-1},Y^{(2),k-1}\right)+I\left(Y_{k}^{\left(1\right)};Y^{(2),k-1}|Y^{(1),k-1}\right)+I\left(Y_{k}^{\left(2\right)};Y^{(1),k-1}|Y^{(2),k-1}\right). \end{array}$$

Consider the first time step of k=1:

(A14) $$\begin{array}{cc} I\left(X_{1};Y_{1}^{\left(1\right)},Y_{1}^{\left(2\right)}|\emptyset \right)+I\left(Y_{1}^{\left(2\right)};Y_{1}^{\left(1\right)}|\emptyset ,\emptyset \right)+I\left(Y_{1}^{\left(1\right)};\emptyset |\emptyset \right)+I\left(Y_{1}^{\left(2\right)};\emptyset |\emptyset \right)\; & =I\left(X_{1};Y_{1}^{\left(1\right)},Y_{1}^{\left(2\right)}\right)\\ & \;+I\left(Y_{1}^{\left(2\right)};Y_{1}^{\left(1\right)}\right) \end{array}$$

Now:

(A15) $$\begin{array}{cc} I\left(X_{1};Y_{1}^{\left(1\right)},Y_{1}^{\left(2\right)}\right)+I\left(Y_{1}^{\left(2\right)};Y_{1}^{\left(1\right)}\right)\; & \overset{\left(a\right)}{\geq }I\left(X_{1};Y_{1,G}^{\left(1\right)},Y_{1,G}^{\left(2\right)}\right)+I\left(Y_{1}^{\left(2\right)};Y_{1}^{\left(1\right)}\right)\\ \; & =I\left(X_{1};Y_{1,G}^{\left(1\right)}\right)+I\left(X_{1};Y_{1,G}^{\left(2\right)}\right)+I\left(Y_{1,G}^{\left(1\right)};Y_{1,G}^{\left(2\right)}|X\right) \end{array}$$

(A16) $$\begin{array}{cc} \; & \;-I\left(Y_{1,G}^{\left(1\right)};Y_{1,G}^{\left(2\right)}\right)+I\left(Y_{1}^{\left(1\right)};Y_{1}^{\left(2\right)}\right), \end{array}$$

where subscript *G* denotes Gaussian random variables, and (a) follows from ([46] Theo. 1.8.6) with equality if $Y^{\left(1\right)},Y^{\left(2\right)}$ are jointly Gaussian with *X*. The last equality follows from the identity:

$$I\left(A;B,C\right)=I\left(A;B\right)+I\left(A;C\right)+I\left(B;C|A\right)-I\left(B;C\right).$$

We recall that the noncausal and arbitrary-delay, lower bound of El-Gamal and Cover [11] is tight for scalar IID Gaussian sources. In the first time step, we may regard $X_{1}$ as a sample from a white Gaussian process with distribution $N(0,\mathrm{Var}\left[X_{1}\right])$. Therefore, the causal and zero-delay coding rate of $X_{1}$ can never do better than the lower bound of El-Gamal and Cover. We recognize the first three terms in Equation (A16) as the El-Gamal and Cover region. Thus, the difference $I\left(Y_{1}^{\left(1\right)};Y_{1}^{\left(2\right)}\right)-I\left(Y_{1,G}^{\left(1\right)};Y_{1,G}^{\left(2\right)}\right)$ can never be negative, since this would violate the tightness of the lower bound. Therefore:

(A17) $$\begin{array}{cc} I\left(X_{1};Y_{1}^{\left(1\right)},Y_{1}^{\left(2\right)}\right)+I\left(Y_{1}^{\left(2\right)};Y_{1}^{\left(1\right)}\right)\; & \geq I\left(X_{1};Y_{1,G}^{\left(1\right)}\right)+I\left(X_{1};Y_{1,G}^{\left(2\right)}\right)+I\left(Y_{1,G}^{\left(1\right)};Y_{1,G}^{\left(2\right)}|X\right) \end{array}$$

(A18) $$\begin{array}{cc} \; & =I\left(X_{1};Y_{1,G}^{\left(1\right)},Y_{1,G}^{\left(2\right)}\right)+I\left(Y_{1,G}^{\left(2\right)};Y_{1,G}^{\left(1\right)}\right) \end{array}$$

with equality if $Y_{1}^{\left(1\right)},Y_{1}^{\left(2\right)}$ are jointly Gaussian.

Now, for the next time step of k=2, we consider:

(A19) $$\begin{array}{c} I\left(X^{2};Y_{2}^{\left(1\right)},Y_{2}^{\left(2\right)}|Y_{1}^{\left(1\right)},Y_{1}^{\left(2\right)}\right)+I\left(Y_{2}^{\left(2\right)};Y_{2}^{\left(1\right)}|Y_{1}^{\left(1\right)},Y_{1}^{\left(2\right)}\right)+I\left(Y_{2}^{\left(1\right)};Y_{1}^{\left(2\right)}|Y_{1}^{\left(1\right)}\right)+I\left(Y_{2}^{\left(2\right)};Y_{1}^{\left(1\right)}|Y_{1}^{\left(2\right)}\right). \end{array}$$

However, we just showed that to be optimal in the first step $Y_{1}^{\left(1\right)},Y_{1}^{\left(2\right)}$ should be jointly Gaussian. Therefore, under the sequential greedy condition, we have that:

(A20) $$\begin{array}{cc} \; & I\left(X^{2};Y_{2}^{\left(1\right)},Y_{2}^{\left(2\right)}|Y_{1}^{\left(1\right)},Y_{1}^{\left(2\right)}\right)+I\left(Y_{2}^{\left(2\right)};Y_{2}^{\left(1\right)}|Y_{1}^{\left(1\right)},Y_{1}^{\left(2\right)}\right)+I\left(Y_{2}^{\left(1\right)};Y_{1}^{\left(2\right)}|Y_{1}^{\left(1\right)}\right)+I\left(Y_{2}^{\left(2\right)};Y_{1}^{\left(1\right)}|Y_{1}^{\left(2\right)}\right)\\ = & I\left(X^{2};Y_{2}^{\left(1\right)},Y_{2}^{\left(2\right)}|Y_{1,G}^{\left(1\right)},Y_{1,G}^{\left(2\right)}\right)+I\left(Y_{2}^{\left(2\right)};Y_{2}^{\left(1\right)}|Y_{1,G}^{\left(1\right)},Y_{1,G}^{\left(2\right)}\right)+I\left(Y_{2}^{\left(1\right)};Y_{1,G}^{\left(2\right)}|Y_{1,G}^{\left(1\right)}\right)+I\left(Y_{2}^{\left(2\right)};Y_{1,G}^{\left(1\right)}|Y_{1,G}^{\left(2\right)}\right) \end{array}$$

Let:

(A21) $$\begin{array}{cc} W^{2} & ≜X^{2}-E\left[X^{2}|Y_{1,G}^{\left(1\right)},Y_{1,G}^{\left(2\right)}\right] \end{array}$$

(A22) $$\begin{array}{cc} U_{2}^{\left(i\right)} & ≜Y_{2}^{\left(i\right)}-E\left[Y_{2}^{\left(i\right)}|Y_{1,G}^{\left(1\right)},Y_{1,G}^{\left(2\right)}\right],\;i=1,2 \end{array}$$

be the residuals for the MMSE predictions of $X^{2},\;Y_{2}^{\left(1\right)},\;Y_{2}^{\left(2\right)}$ given $Y_{1,G}^{\left(1\right)},Y_{1,G}^{\left(2\right)}$. Then, considering the first two terms in Equation (A20), we have that:

(A23) $$\begin{array}{cc} \; & I\left(X^{2};Y_{2}^{\left(1\right)},Y_{2}^{\left(2\right)}|Y_{1,G}^{\left(1\right)},Y_{1,G}^{\left(2\right)}\right)+I\left(Y_{2}^{\left(2\right)};Y_{2}^{\left(1\right)}|Y_{1,G}^{\left(1\right)},Y_{1,G}^{\left(2\right)}\right)\\ = & I\left(W^{2};U_{2}^{\left(1\right)},U_{2}^{\left(2\right)}|Y_{1,G}^{\left(1\right)},Y_{1,G}^{\left(2\right)}\right)+I\left(U_{2}^{\left(2\right)};U_{2}^{\left(1\right)}|Y_{1,G}^{\left(1\right)},Y_{1,G}^{\left(2\right)}\right) \end{array}$$

By the orthogonality principle, the residuals of the MMSE estimators are uncorrelated with the conditioning variables, $Y_{1,G}^{\left(1\right)},Y_{1,G}^{\left(2\right)}$ [34]. Therefore, since $X^{2}$ is Gaussian, $W^{2}$ is Gaussian and independent of $Y_{1,G}^{\left(1\right)},Y_{1,G}^{\left(2\right)}$. Furthermore, by the conditional residual independence assumption, $U_{2}^{\left(i\right)},\;i=1,2$ are assumed independent of $Y_{1,G}^{\left(1\right)},Y_{1,G}^{\left(2\right)}$, which is true for Gaussian $Y_{2}^{\left(i\right)}$. Thus:

(A24) $$\begin{array}{c} I\left(W^{2};U_{2}^{\left(1\right)},U_{2}^{\left(2\right)}|Y_{1,G}^{\left(1\right)},Y_{1,G}^{\left(2\right)}\right)+I\left(U_{2}^{\left(2\right)};U_{2}^{\left(1\right)}|Y_{1,G}^{\left(1\right)},Y_{1,G}^{\left(2\right)}\right)=I\left(W^{2};U_{2}^{\left(1\right)},U_{2}^{\left(2\right)}\right)+I\left(U_{2}^{\left(2\right)};U_{2}^{\left(1\right)}\right). \end{array}$$

Using the same technique and arguments as in the first time step, we can lower bind these two terms:

(A25) $$\begin{array}{c} I\left(W^{2};U_{2}^{\left(1\right)},U_{2}^{\left(2\right)}\right)+I\left(U_{2}^{\left(2\right)};U_{2}^{\left(1\right)}\right)\geq I\left(W^{2};U_{2,G}^{\left(1\right)},U_{2,G}^{\left(2\right)}\right)+I\left(U_{2,G}^{\left(2\right)};U_{2,G}^{\left(1\right)}\right) \end{array}$$

with equality if $U_{2}^{\left(1\right)},U_{2}^{\left(2\right)}$ are jointly Gaussian, or equivalently when $Y_{2}^{\left(1\right)},Y_{2}^{\left(2\right)}$ are jointly Gaussian.

Now consider the last two terms in Equation (A20):

(A26) $$\begin{array}{cc} I\left(Y_{2}^{\left(1\right)};Y_{1,G}^{\left(2\right)}|Y_{1,G}^{\left(1\right)}\right)+I\left(Y_{2}^{\left(2\right)};Y_{1,G}^{\left(1\right)}|Y_{1,G}^{\left(2\right)}\right) & =I\left(Y_{2}^{\left(1\right)}-E\left[Y_{2}^{\left(1\right)}|Y_{1,G}^{\left(1\right)}\right];Y_{1,G}^{\left(2\right)}-E\left[Y_{1,G}^{\left(2\right)}|Y_{1,G}^{\left(1\right)}\right]|Y_{1,G}^{\left(1\right)}\right)\\ \; & \;+I\left(Y_{2}^{\left(2\right)}-E\left[Y_{2}^{\left(2\right)}|Y_{1,G}^{\left(2\right)}\right];Y_{1,G}^{\left(1\right)}-E\left[Y_{1,G}^{\left(1\right)}|Y_{1,G}^{\left(2\right)}\right]|Y_{1,G}^{\left(2\right)}\right)\\ \; & \overset{\left(b\right)}{=}I\left(Y_{2}^{\left(1\right)}-E\left[Y_{2}^{\left(1\right)}|Y_{1,G}^{\left(1\right)}\right];Y_{1,G}^{\left(2\right)}-E\left[Y_{1,G}^{\left(2\right)}|Y_{1,G}^{\left(1\right)}\right]\right)\\ \; & \;+I\left(Y_{2}^{\left(2\right)}-E\left[Y_{2}^{\left(2\right)}|Y_{1,G}^{\left(2\right)}\right];Y_{1,G}^{\left(1\right)}-E\left[Y_{1,G}^{\left(1\right)}|Y_{1,G}^{\left(2\right)}\right]\right), \end{array}$$

where (b) follows since we assume conditional prediction residual independence of the MMSE predictors. Since the residuals on the right side of the mutual informations are Gaussian, the mutual information is minimized if the residuals on the left, $Y_{2}^{\left(i\right)}-E\left[Y_{2}^{\left(i\right)}|Y_{1,G}^{\left(i\right)}\right]$, i=1,2, are Gaussian—that is, when $Y_{2}^{\left(i\right)},\;i=1,2$ are Gaussian. Thus:

(A27) $$\begin{array}{c} I\left(X^{2};Y_{2}^{\left(1\right)},Y_{2}^{\left(2\right)}|Y_{1}^{\left(1\right)},Y_{1}^{\left(2\right)}\right)+I\left(Y_{2}^{\left(2\right)};Y_{2}^{\left(1\right)}|Y_{1}^{\left(1\right)},Y_{1}^{\left(2\right)}\right)+I\left(Y_{2}^{\left(1\right)};Y_{1}^{\left(2\right)}|Y_{1}^{\left(1\right)}\right)+I\left(Y_{2}^{\left(2\right)};Y_{1}^{\left(1\right)}|Y_{1}^{\left(2\right)}\right)\\ \geq I\left(X^{2};Y_{2,G}^{\left(1\right)},Y_{2,G}^{\left(2\right)}|Y_{1,G}^{\left(1\right)},Y_{1,G}^{\left(2\right)}\right)+I\left(Y_{2,G}^{\left(2\right)};Y_{2,G}^{\left(1\right)}|Y_{1,G}^{\left(1\right)},Y_{1,G}^{\left(2\right)}\right)+I\left(Y_{2,G}^{\left(1\right)};Y_{1,G}^{\left(2\right)}|Y_{1,G}^{\left(1\right)}\right)+I\left(Y_{2,G}^{\left(2\right)};Y_{1,G}^{\left(1\right)}|Y_{1,G}^{\left(2\right)}\right) \end{array}$$

with equality if $Y_{2}^{\left(1\right)},Y_{2}^{\left(2\right)}$ are jointly Gaussian, given that $Y_{1}^{\left(1\right)},Y_{1}^{\left(2\right)}$ are jointly Gaussian, which they are by the sequential greedy assumption. The result now follows by induction on *k*, dividing by *n*, and taking the limit as n→∞. □

## Notation

Symbol	Description
R	The set of real numbers
N	The set of natural numbers
$N^{n}$	The set $\left\{1,\ldots ,n\right\}$, n∈N
*X*	Random variable
X	Alphabet for the random variable *X*
$X^{n}$	Sequence of n∈N random variables $\left(X_{1},\ldots ,X_{n}\right)$
$x^{n}$	Sequence of n∈N random variable realizations, where $x^{n}\in X^{n}$
$X^{n}$	$\times_{k=1}^{n}X_{k}$, with $X_{k}=X$
X⫫Y	For independent random variables, X,Y
X−Y−Z	When the random variables X,Y,Z form a Markov chain in that order
	i.e., when P(X,Z|Y)=P(X|Y)P(Z|Y)
${X|}_{W}-Y{{|}_{W}-Z|}_{W}$	If the Markov chain is conditioned upon *W*
